# Metabolic, structural, and proteomic changes in *Candida albicans* cells induced by the protein-carbohydrate fraction of *Dendrobaena veneta* coelomic fluid

**DOI:** 10.1038/s41598-021-96093-1

**Published:** 2021-08-18

**Authors:** Marta J. Fiołka, Paulina Czaplewska, Sylwia Wójcik-Mieszawska, Aleksandra Lewandowska, Kinga Lewtak, Weronika Sofińska-Chmiel, Tomasz Buchwald

**Affiliations:** 1 Department of Immunobiology, Institute of Biological Sciences, Maria Curie-Skłodowska University, Akademicka 19, 20-033 Lublin, Poland; 2 Intercollegiate Faculty of Biotechnology, University of Gdansk and Medical University of Gdansk, Gdansk, Poland; 3 Department of Cell Biology, Institute of Biological Sciences, Maria Curie-Skłodowska University, Lublin, Poland; 4 Analytical Laboratory, Institute of Chemical Sciences, Maria Curie-Skłodowska University, Lublin, Poland; 5 Faculty of Materials Science and Technical Physics, Institute of Materials Research and Quantum Engineering, Poznan University of Technology, Poznań, Poland

**Keywords:** Biotechnology, Cancer, Drug discovery, Microbiology

## Abstract

The isolated protein-polysaccharide fraction (AAF) from the coelomic fluid of *Dendrobaena veneta* earthworm shows effective activity against *Candida albicans* yeast. Fungal cells of the clinical strain after incubation with the active fraction were characterized by disturbed cell division and different morphological forms due to the inability to separate the cells from each other. Staining of the cells with acridine orange revealed a change in the pH of the AAF-treated cells. It was observed that, after the AAF treatment, the mitochondrial DNA migrated towards the nuclear DNA, whereupon both merged into a single nuclear structure, which preceded the apoptotic process. Cells with a large nucleus were imaged with the scanning electron cryomicroscopy (Cryo-SEM) technique, while enlarged mitochondria and the degeneration of cell structures were shown by transmission electron microscopy (TEM). The loss of the correct cell shape and cell wall integrity was visualized by both the TEM and SEM techniques. Mass spectrometry and relative quantitative SWATH MS analysis were used to determine the reaction of the *C. albicans* proteome to the components of the AAF fraction. AAF was observed to influence the expression of mitochondrial and oxidative stress proteins. The oxidative stress in *C. albicans* cells caused by the action of AAF was demonstrated by fluorescence microscopy, proteomic methods, and XPS spectroscopy. The secondary structure of AAF proteins was characterized by Raman spectroscopy. Analysis of the elemental composition of AAF confirmed the homogeneity of the preparation. The observed action of AAF, which targets not only the cell wall but also the mitochondria, makes the preparation a potential antifungal drug killing the cells of the *C. albicans* pathogen through apoptosis.

## Introduction

It is astonishing that such an inconspicuous invertebrate as the earthworm used as baits for fish has gained so much importance in biomedical research^1^. However, this can be understood considering the importance of earthworms in Far Eastern cultures^2–4^. Preparations from earthworms are recognized as effective anti-inflammatory, analgesic, antipyretic, and anticancer agents in oriental medicine^5,6^. Earthworms live in an environment with high richness of various microorganisms and play an important role in regulating the microbial composition of this environment. As shown by Khomyakov and collaborators^7^, antimicrobial compounds do not originate from soil microorganisms that enter the gastrointestinal tract of earthworms, but they are produced in their body. Among the beneficial or neutral soil microorganisms, there are pathogens, many of which are pathogenic to humans in certain conditions. To survive in the environment, earthworms had to develop a number of defense mechanisms in the course of evolution, such as the production of antimicrobial substances, which we try to use in the fight against human diseases. Earthworms have the potential to offer many health benefits to humans and pose no difficulties to researchers in terms of the cost and ethics of animal research^1^. Thus, bioprospection in relation to earthworms is still an evolving field.

Since the end of the twentieth century, the number of infections caused by fungi has increased significantly. The phenomenon is observed especially in intensive care units in Europe and the USA^8–11^, where *Candida albicans* is most frequently responsible for fungal infections. It can induce superficial or systemic candidiasis. *C. albicans* is the most often isolated pathogen in samples from patients with urinary tract infections and vaginitis^12^ and is responsible for 70% of invasive candidiasis, which can be fatal in up to 79% of cases^10,13^. The cause of the increased number of cases of fungal diseases is the increasing number of susceptible subjects whose immune balance has been disturbed by invasive surgery or a long stay in the intensive care unit. Other factors that increase the risk of infection are treatment with chemotherapy, immunosuppressants, or antibiotics as well as HIV infection and a very young or old age^13–15^. Surgical interventions and insertion such of medical devices as joint prostheses, catheters, and pacemakers are also a risk factor^14,16,17^. At the same time, the number of cases is three times higher in burn wards than in other intensive care wards^15^.

*C. albicans* can cause life-threatening infections due to its ability to alter its growth model. In normal conditions, this microorganism grows as a commensal on the surface of the mucous membranes of the mouth, vagina, and skin and as part of the intestinal microflora^14,18^. The immune system keeps it unicellular, preventing excessive proliferation and development of infection^19^. When the balance is disturbed, *C. albicans* multiplication is no longer controlled by the immune system. The cells begin to produce pseudo-hyphae, mycelium, or even biofilm and break through the host's defense barriers. The biofilm is extremely difficult to remove. It can develop on both natural and synthetic surfaces. It is also a reservoir of fungal cells, which when released into the bloodstream create new outbreaks of candidiasis or serious blood infections in the organism^11,14,17^. The antibiotics used in candidiasis treatment have many side effects, which are especially dangerous to patients with a weakened immune system by e.g., chemotherapy or HIV infection. The side effects of these medications range from nausea, diarrhea, and rashes to severe hormonal imbalances and liver damage^20^. It is risky to administer these types of antibiotics to seriously ill subjects^21,22^. There are also an increasing number of *Candida* strains that are resistant to conventionally used antibiotics, e.g., azoles or echinocandins^10,14^.

The search for new effective preparations for the treatment of candidiasis is extremely important due to the constantly growing number of *C. albicans* strains with resistance to commonly used antifungal antibiotics and the growing group of subjects that are susceptible to fungal infections^23^. To date, the research into the antimicrobial effects of earthworms has focused mainly on pastes, extracts, and powders prepared from these invertebrates and their properties^4,24–29^.

Our research resulted in isolation of the protein-polysaccharide fraction, which is a chemically homogeneous complex effectively inhibiting the growth of *C. albicans* cells, significantly decreasing the metabolic activity of fungal cells, and leading to their death. Moreover, the study did not show any undesirable effects of cytotoxicity and endotoxicity to normal human fibroblasts^30^. The fraction exhibits antitumor activity against A549 lung cancer^31^ and human colon adenocarcinoma cells^32^. The properties of the active fraction make it a promising potential agent for the treatment of candidiasis; therefore, its mechanism of action should be thoroughly analyzed. The aim of the research was to provide new biological and chemical data on the mechanisms of action of the active coelomic fluid fraction (AAF) against the cells of a clinical *C. albicans* strain.

## Materials and methods

### Earthworms

The earthworm *Dendrobaena veneta* was the animal research model. The invertebrates were reared in laboratory conditions at the Department of Immunobiology, Maria Curie-Skłodowska University in Lublin, Poland. The earthworms were grown in 3L containers filled with compost soil at 70–80% humidity and a temperature of approx. 20 °C. The containers with the animals were kept in total darkness. The animals were fed boiled vegetables with the addition of pure cellulose twice a week. Only adult individuals were selected for the experiments.

### Extraction of the active fraction (AAF) from earthworm coelomic fluid

The earthworms were placed in containers filled with moist lignin for 24 h to clean their gastrointestinal tract. The animals were then washed individually and blotted dry. The method of electrical stimulation (4.5 V) was used to collect coelomic fluid (CF). Then, CF was taken up to 0.9% NaCl from groups of 10 (1500 µl per group). CF together with the coelomocytes was centrifuged at 6000×*g* for 10 min at 4 °C. The separated supernatant from the coelomocytes was filtered through 0.22 µm Millipore filters. The cell-free CF was incubated for 10 min at 70 °C to remove cytotoxic properties. Next, it was transferred to a cellulose membrane bag with a cut-off of 12–14 kDa. The samples were dialyzed in water for 24 h at 4 °C. After dialysis, the fraction (AAF) was transferred to Eppendorf tubes for lyophilization. AAF was stored at − 20 °C. The Bradford assay (Bio-Rad) was used to determine the protein concentration in the preparation^33^.

### Microorganisms and preparation for microscopy

A clinical isolate of a *C. albicans* wild type strain gifted by Prof. A. Kędzia, Department of Oral Microbiology, Medical University of Gdańsk, was used for microbiological analysis. The effect of AAF on the *C. albicans* strain—a wild-type clinical isolate was analyzed. The strain was pre-grown on standard YPD rich medium at 28 °C for 24 h. The experiments were performed using YPD poor liquid medium (0.1% yeast extract, 0.2% glucose, 0.05% peptone). AAF at final concentrations of 25, 50, and 100 µg mL^−1^ was added to 150 µL of YPD poor medium containing the fungal cell culture (10^7^ CFU from the logarithmic growth phase). Streptomycin sulfate (Sigma) at final concentration of 0.17 mg mL^−1^ was then added to the *C. albicans* suspension and supplemented with YPD poor medium to a final volume of 250 µL. The samples were incubated on a shaker for 48 h at 37 °C. After this time, the antifungal activity of AAF was analyzed with biological and chemical methods.

### Imaging fungal cells after acridine orange staining

Acridine orange (AO) stains both live and dead cells. The fluorochrome is able to cross the damaged membrane and accumulate in dead cells due to specific binding to nucleic acids. An acridine orange solution at 0.1 mg mL^−1^ was used to stain *C. albicans* cells. Cultured cells were harvested by centrifugation (at 2200 g, for 10 min, at room temperature), rinsed twice, and suspended in Ringer's solution. The fluorochrome was added in a 1:1 ratio to the cell suspension of the control culture and the AAF-treated culture, and the samples were then incubated for 10 min in the dark at room temperature. After this time, 2 µL of the suspension were placed on a microscope slide and observed using a Zeiss/LEO 912AB microscope at × 1000 magnification. The microscopic parameters were as follows: excitation wavelength 502 nm, emission wavelength 526 nm, filter wavelength 470 nm^34^.

### Imaging fungal cells after Hoechst and propidium iodide staining

Control *C. albicans* cells and cells incubated with AAF were stained with a mixture of fluorescent dyes: Hoechst 33342 (Sigma) and propidium iodide (Sigma)^30^. The staining mixture was prepared and added in a 1:1 volume to the *C. albicans* cell suspension, followed by incubation for 5 min at 37 °C in the dark. The fungal cells were analyzed under a Zeiss LSM 5 Pascal fluorescence microscope. Normal cell nuclei were stained blue, whereas apoptotic cells were strongly fluorescent. Cells with pink, fluorescent nuclei were interpreted as necrotic. The Hoechst dye is commonly used for staining not only cell nuclei but also mitochondria by visualizing DNA in fluorescence microscopy. Mitochondrial DNA emits green fluorescence in the images.

### Oxidative stress assay

For the oxidative stress assay, the fungal cultures were prepared according to the procedure described in “Microorganisms and preparation for microscopy” A positive control of oxidative activity in the yeast cells was prepared by incubating *C. albicans* cells with H_2_O_2_ at a final concentration of 100 µM in the same conditions as with AAF. To assess the levels of reactive oxygen species (ROS) in AAF-treated *C. albicans* cells, the 2′,7′-dichlorofluorescin diacetate (H_2_DCF-DA) assay was used^35,36^. This method is based on the action of cellular esterase on H_2_DCF-DA releasing an intermediate polar form H_2_DCF, which reacts with ROS to form a highly fluorescent product, i.e., 2',7'-dichlorofluorescein (DCF)^37^. The H_2_DCF-DA reagent (Sigma-Aldrich D6883) was dissolved immediately before use in anhydrous DMSO to obtain a 10 mM stock solution. 2 μL of the reconstituted dye was diluted in 1 mL of PBS to give a final concentration of 20 μM. Fungal cells were centrifuged at 6000×*g* for 10 min, washed in PBS buffer pH 7.4, and centrifuged again. The pellet was incubated in H_2_DCF-DA (20 μM) for 15 min in the dark at 37 °C. The stained cells were placed on microscopic slides and captured immediately using a Zeiss/LEO 912AB microscope at 1000 × magnification. The excitation and emission wavelengths used for dichlorofluorescin diacetate were 504 nm and 524 nm, respectively. The experiment was repeated three times.

### Scanning Electron Cryomicroscopy (Cryo-SEM) analysis

Cells from the control *C. albicans* culture and those incubated with AAF were centrifuged at 6000×*g* for 10 min. The supernatant was then discarded and 200 µL of GH solution (glucose, Na-HEPES, sterile water) were added to the dense cell suspension. The samples were re-centrifuged in the same conditions, and the supernatant was discarded again. The fungal cells in a small amount of the GH solution were applied to the transfer and placed in the sublimation chamber. The cooling process was carried out for 12 min at − 92 °C. Then, the samples were transferred to the preparation chamber, where they were broken using a special blade. *C. albicans* cells were then imaged with a scanning electron microscope (ZEISS Ultra Plus Field Emission microscope). The images were taken at a magnification of 30.000 × and at electron high tension (EHT) 5 kV.

### Transmission Electron Microscopy (TEM) analysis

*Candida albicans* cells were fixed with 4% glutaraldehyde in 0.1 M cacodyl buffer (pH 7.2) with the addition of 0.8 M sorbitol. Then, the fungal cells were re-fixed in a 1.5% potassium permanganate solution. The preparations were contrasted en-bloc with 1% uranyl acetate and dehydrated with ethanol series, supersaturated, and embedded in LR White resin^38^. Ultrathin sections were observed using a Tecnai G2 T20 X-TWIN(FEI) transmission electron microscope.

### Scanning Electron Microscopy (SEM) analysis

*Candida albicans* yeast cells from the control culture and those incubated with AAF with a protein concentration of 100 µg mL^−1^ were fixed with a glutaraldehyde solution at pH 7. The cells were then rinsed with sodium phosphate buffer at pH 7, flooded with a 1.5% OsO_4_ solution, and incubated at room temperature for 30 min. Next, they were treated with acetone solutions with increasing concentrations of 30, 50, 70, and 100% to dehydrate. The cell suspensions in acetone were spotted on SEM tables and dried in the presence of silica gel beads for 24 h at room temperature. The samples were then sputtered with gold using a K550X sputter coater (Quorum Technologies). The *C. albicans* cells were imaged using a Vega 3 scanning electron microscope (Tescan) with a 30 kV electron beam voltage.

### MED-FASP digestion of AAF

The AAF sample was treated with lysis buffer (100 mM Tris–HCl, 1% SDS, 50 mM DTT), according to the standard MED-FASP protocol^39^. The protein concentration was established by measuring absorbance at 280 nm (Multiskan Thermo) using a μDrop plate. 100 µg of proteins were digested on 10 kDa Microcons (Merck-Millipore) successively by Lys-C, trypsin, and chymotrypsin. The fractions collected after the centrifugation were subjected to the final clean up with StageTips according to the protocol described by Rappsilber and collaborators^40^. For each desalting step, 10 µg of the peptides were taken and desalted on StageTip (3 layers of 3 M Empore C18 exchange discs in each tip). Fragmentation spectra were recorded on the Triple TOF 5600 + (Sciex) spectrometer (SCIEX, Framingham, MA) coupled with the EkspertMicroLC 200 Plus System (Eksigent, Redwood City, CA). Registration was performed in the data-dependent acquisition (DDA) mode, and the protein identification was conducted in the Peaks Studio program against the Annelida database (Uniprot).

### Lysis of *C. albicans* cells

All samples of *C. albicans* cells were lysed according to the procedure presented by von der Haar^41^. In brief, the cells were suspended in a solution of 0.1 M NaOH, 0.05 M EDTA, 2% of SDS, and 2% of β-mercaptoethanol and heated in a thermoblock at 90 °C for 10 min. The solutions were then acidified by adding 4 M acetic acid and reheating at 90 °C for additional 10 min. After cooling, cold acetone was added to each Eppendorf to precipitate proteins and kept for 2 h in the freezer. Next, the solutions were centrifuged and decanted to give a pre-purified protein fraction, freeze-dried to remove water, and stored at − 20 °C for further experiments.

### AAF immobilization of CNBr sepharose

The AAF-sepharose column was prepared following the protocol proposed by Kavran and Leahy^42^. 100 mg of CNBr sepharose were used for preparation of one column and the same amount was used for the control. A sample of lyophilized AAF was dissolved in coupling buffer (1 mg mL^−1^), added to the activated matrix, and mixed for 2 h. Next, the column was washed and blocked according to the protocol. The control column was directly treated with blocking solutions. The concentrations of the starting AAF solution and unbound proteins were checked by measuring the absorbance at 280 nm. The coupling efficiency was around 75%. Ready columns (AAF and control one) were washed and kept in storage buffer at 4 °C.

### Affinity chromatography

The lyophilized sample of *C. albicans* proteins obtained after the basic lysis of the cells was dissolved in PBS. 100 μg of the proteins was added to the AAF column and the control one and mixed for 2 h at room temperature. Next, the supernatant was collected, and both columns were washed with five volumes of phosphate buffer; the last ml of the wash was collected. Elution of bound proteins was performed with a 0.1% TFA/water solution. All fractions (supernatant, wash, and elution) from the AAF-column and the control were collected and lyophilized for further proteomic identification.

### Quantitative analysis

#### FASP digestion of AAF-treated *C. albicans* cells

*C. albicans* cells treated with different AAF concentrations (25, 50, 100 µg mL^−1^) were lysed, and released proteins were prepared as described above. Before digestion, all samples were dissolved in a urea-containing solution (8 M urea in 0.1 M Tris–HCl pH 8.5). The protein concentration was established by measuring absorbance at 280 nm (Multiskan Thermo) using a μDrop plate. 100 µg of protein from each of the samples were digested separately with trypsin on 10 kDa Microcons (Merck-Millipore) according to the standard FASP protocol and prepared for mass spectrometry analysis^43^. Final clean up preceding mass spectrometry measurements was done with StageTips according to the protocol described by Rappsilber and collaborators^40^. For each desalting step, 10 µg of the peptide were taken and desalted on StageTip (3 layers of 3 M Empore C18 exchange discs in each tip).

#### Quantitative LC–MS/MS analysis

##### Liquid chromatography and mass spectrometry

The LC–MS/MS analysis was performed using a Triple TOF 5600 + mass spectrometer (SCIEX, Framingham, MA) coupled with the EkspertMicroLC 200 Plus System (Eksigent, Redwood City, CA). All chromatographic separations were performed on a ChromXP C18CL column (3 μm, 120 Å, 150 × 0.3 mm). The chromatographic gradient for each DDA and SWATH run was 7–35% B (solvent A: 99.9% aqueous solution, 0.1% formic acid; solvent B: 99.9% acetonitrile, 0.1% formic acid) for 60 min. The whole system was controlled by the SCIEX Analyst TF 1.7.1 software. Shotgun experiments in the data-dependent acquisition were acquired in a precursor mass range of 400–1200 Da for a maximum of 20 candidate ions per cycle with exclusion for 5 s after 2 occurrences within an accumulation time of 100 ms. The precursor ion scan was followed by the product ion scan in the range of 100–1800 Da within an accumulation time of 50 ms.

##### SWATH mass spectrometry experiments

All samples were acquired in triplicates. The experiments were performed in a looped product ion mode. A set of 25 transmission windows (variable width) was constructed using the swath turner tool^44^ with the equalized frequency approach on the data acquired from the DDA measurements of the control sample. It covered the precursor mass range of 400–1200 m/z. The collision energy for each window was calculated for + 2 to + 5 charged ions centered upon the window with a spread of 2. The SWATH-MS1 survey scan was acquired in a high sensitivity mode in the range of 400–1200 Da at the beginning of each cycle with the accumulation time of 50 ms. SWATH-MS/MS spectra were collected in the range of 100–1800 m/z during 40 ms accumulation time high sensitivity product ion scans, which resulted in the total cycle time of 1.1 s.

#### Data analysis

A database search of the DDA measurements was performed with ProteinPilot 4.5 software (Sciex) using the Paragon algorithm against the UNIPROT *C. albicans* database with an automated false discovery rate and standard parameters^45,46^. Next, a spectral library was created with the group file data processing in PeakView 2.2 (SCIEX), with parameters described in detail by Lewandowska and collaborators^45^. Files from the SWATH experiments for each sample were downloaded to PeakView software and processed with the previously established library. Resulting data were exported to the .xml file and exported to Marker View software. All data were normalized using the total area sums (TAS) approach, grouped as the wild type and tested samples, and t-tests were performed. The samples were compared to each other. Coefficients of variation (CV%) were calculated, and proteins with a p-value lower than 0.05 with a fold change greater than 10% were considered differentially expressed in the examined samples. The obtained data were analyzed by the STRING server^47,48^ and processed in Cytoscape software^49^.

### X-ray Photoelectron Spectroscopy (XPS) analysis

X-ray photoelectron spectroscopy (XPS) studies were performed using the multichamber UHV system (PREVAC). Spectra were collected using a Scienta SAX-100 x-ray source (Al Kα, 1486.6 eV, 0.8 eV band) equipped with an XM 650 X-ray Monochromator (0.2 eV band) as complementary equipment and a hemispherical Scienta R4000 electron analyzer. The pass energy of the analyzer was set to 200 eV for the survey spectra (with a 500-meV step) and 50 eV for the regions (high-resolution spectra) with a 50-meV step. The pressure in the analysis chamber during the spectra collection was not higher than 2·10^–8^ mbar.

### SEM/Energy Dispersive X-ray Spectroscopy (EDS) analysis

The AAF lyophilizate obtained was imaged with the SEM technique and then its elemental composition was analyzed. The sample was placed on the Al microscope stage and transferred to the microscope sample chamber. The microscope system was equipped with a, EDS spectrometer from EDAX, and EDS measurements were conducted at 30 kV beam voltage. Four different sample locations were spotted, and characteristic spectra were collected for each sample site. The elemental composition expressed as the atomic concentration of the test points was calculated using EDAX.

### Raman Microspectroscopy analysis

The analysis of secondary structure changes on the AAF surface was conducted based on the Raman spectra maps collected by the inVia Renishaw Microscope (Renishaw, UK). Spectroscopic measurements were carried out using a laser emitting 785 nm and 1200 l/mm diffraction grating. The laser beam was automatically focused on the AAF surface using a × 100 microscope objective (Leica Microsystems) with numerical aperture of 0.85. The Raman system was calibrated with the use of the 520.7 cm^−1^ band of the Si internal reference sample. Raman spectra were recorded between 1400 cm^−1^ and 1800 cm^−1^ at room temperature (approx. 23 °C). The intensity of the Raman bands obtained by the curve-fitting process in a range of the Amide I band between 1590 cm^−1^ and 1710 cm^−1^ was used to determine the amount of protein secondary structures (alpha helix, beta sheet, beta turn, and random coil). The curve-fitting process and the position of bands assigned to protein secondary structures was described in detail in our earlier study^30^. A Raman map was collected in the 100 µm × 100 µm area with a step of 10 µm.

### Statistical analysis

Statistical analyses were performed in the Statistica program (Serial number: JPZ904D031610ARACD-W). The normality of the distribution was analyzed with the Shapiro–Wilk test, and the homogeneity of variance was checked with the Levene test. To analyze the significance of differences between the means of individual trials, one-way ANOVA was performed with Tukey’s HSD post-hoc test to assess the significance of the differences. Additionally, using the ω^2^ coefficient (Eq. 1), the strength of the effect induced in *C. albicans* culture by incubation with successive concentrations of the protein-carbohydrate fraction was assessed. A value of the coefficient above 75% indicates a strong effect of the factor on the sample values.

The ω^2^ coefficient:

1 $$ \omega^{{2}} = {{\left\{ {\left[ {{\text{SS}}_{{{\text{EFFECT}}}} - \left( {{\text{df}}_{{{\text{EFFECT}}}} \times {\text{ MS}}_{{{\text{ERROR}}}} } \right)} \right]} \right\}} \mathord{\left/ {\vphantom {{\left\{ {\left[ {{\text{SS}}_{{{\text{EFFECT}}}} - \left( {{\text{df}}_{{{\text{EFFECT}}}} \times {\text{ MS}}_{{{\text{ERROR}}}} } \right)} \right]} \right\}} {\left( {{\text{MS}}_{{{\text{ERROR}}}} + {\text{SS}}_{{{\text{TOTAL}}}} } \right)}}} \right. \kern-\nulldelimiterspace} {\left( {{\text{MS}}_{{{\text{ERROR}}}} + {\text{SS}}_{{{\text{TOTAL}}}} } \right)}} \times {1}00\% $$

## Results

### Fluorescence microscopy imaging of *C. albicans* cells after treatment with AAF

**Figure 1:**
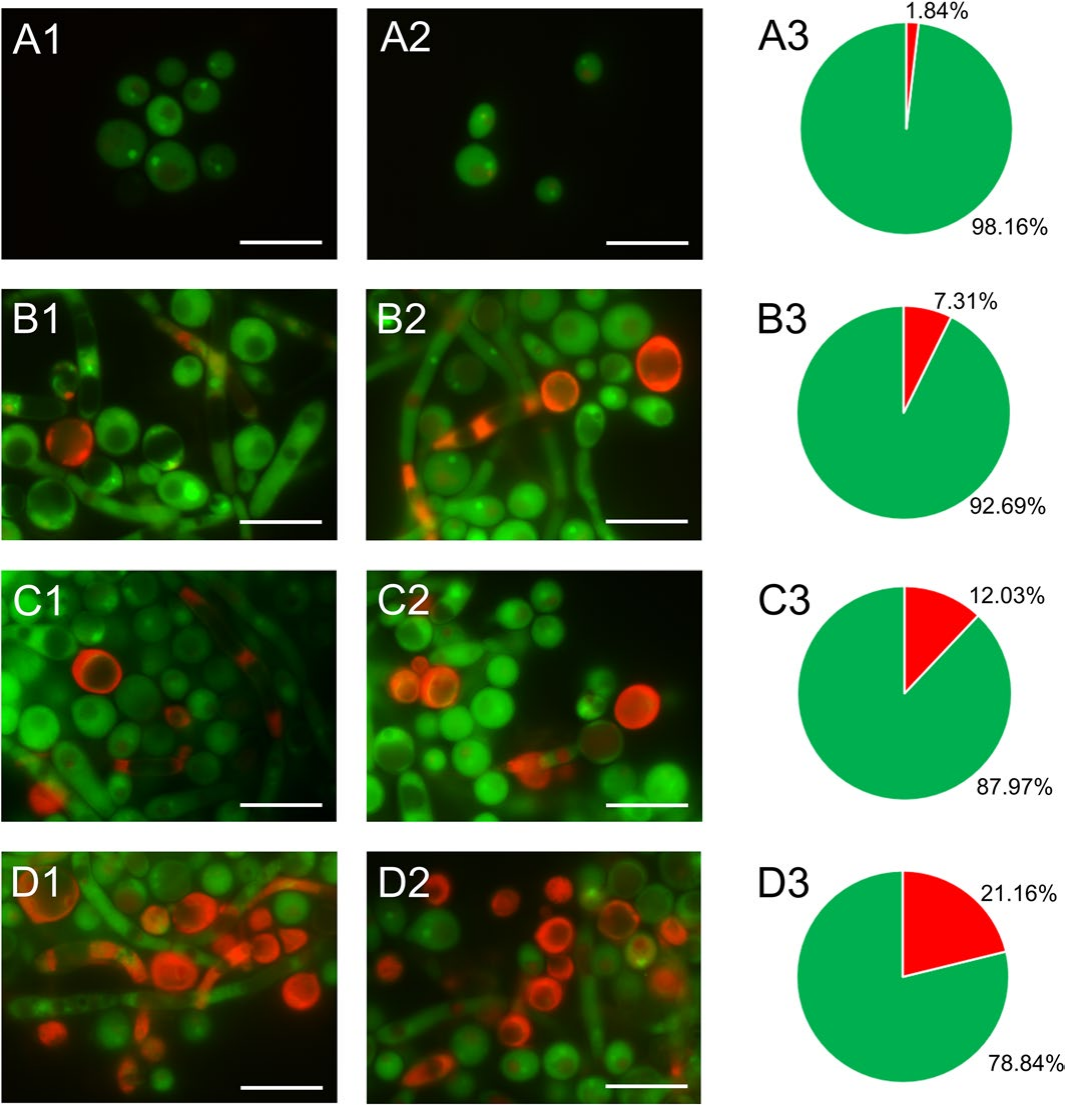
*C. albicans* cells after staining with acridine orange: (**A1**,**A2**) control cells, (**B1**,**B2**) cells treated with AAF at the concentration of 25 µg mL^−1^, (**C1**,**C2**) at the concentration 50 µg mL^−1^, (**D1**,**D2**) at the concentration 100 µg mL^−1^. AO intercalates with DNA and RNA, and the acidic cellular compartments glow orange. The number of cells with the intense orange color increased with the increasing concentration of AAF as shown in diagrams (**A3**, **B3**, **C3**, and **D3**) in this figure. Bars represent 2 µm.

**Figure 2:**
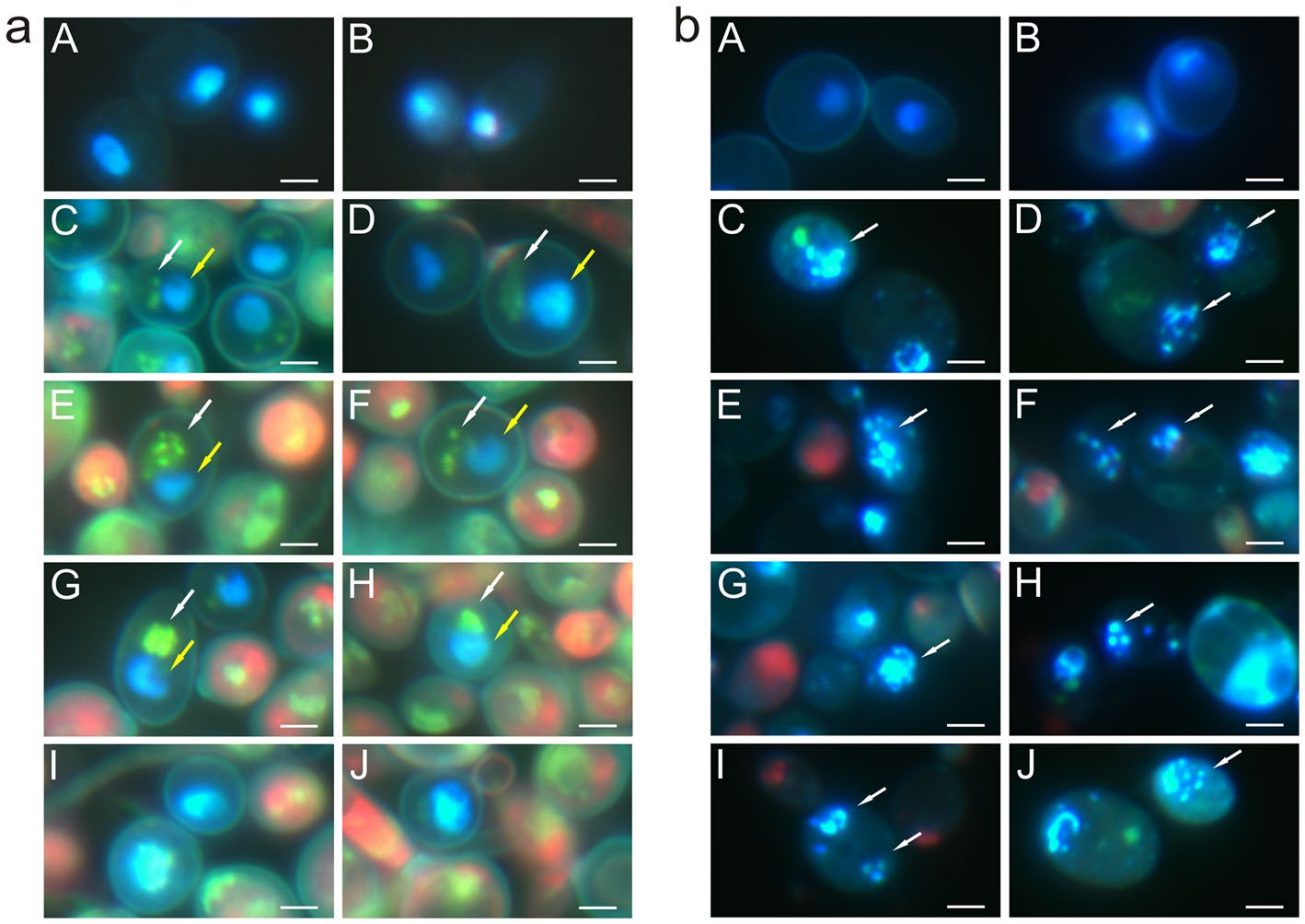
*C. albicans* cells after staining with Hoechst and propidium iodide: (**a**) Migration of mitochondrial DNA into nuclear DNA: (**A**,**B**) control cells, (**C**–**J**) cells treated with AAF (100 µg mL^−1^); (**C**,**D**) blue fluorescent surface of nuclei (yellow arrows), green glowing of mitochondrial DNA (white arrows); (**E**,**F**)migration of mitochondrial DNA to nuclear DNA; (**G**,**H**) fusion of mitochondrial DNA and nuclear DNA into one structure, (**I**,**J**) cells with enlarged nuclei formed after fusion of mitochondrial DNA with nuclear DNA; (**b**) Apoptosis: (**A**,**B**)—control cells, (**C**–**J**) cells treated with AAF (100 µg mL^−1^), cells undergoing the apoptotic process with fragmented genetic material fluorescing intense light blue (marked by white arrows). Bars represent 2 µm.

Staining *C. albicans* cells with fluorescent methods allowed us to distinguish cells with metabolic disorders and altered acidity of the cell environment. Control and AAF-treated *C. albicans* cells were stained with acridine orange. The control cells shown in the images in Fig. 2A1,A2 glow green. After incubation of the fungal cells with AAF at a concentration of 25 µg mL^−1^, orange cells were visible in addition to the green glowing cells (Fig. 1B1,B2). After application of AAF at the concentration of 50 µg mL^−1^, orange glowing cells were seen more frequently in the analyzed microscopic images, as shown in Fig. 1C1,C2. After incubation of the cells with AAF at the concentration of 100 µg mL^−1^, apart from the green cells, whose color differed from that of the control cells, orange cells with a clearly deformed shape and enlarged cells were observed, as can be seen in pictures D1 and D2. The orange glow indicates infiltration of the dye inside the cell and accumulation via specific binding with nucleic acids. The number of cells with the intense orange color increased with the increasing concentration of AAF. Non-viable cells had orange to red colored chromatin with organized structure. The percentage of cells fluorescing green, and orange is shown in diagrams A3-D3 (Fig. 1).

The acridine orange staining was performed in triplicate. Approximately 1400 cells were counted for each concentration in each replication. The Shapiro–Wilk test performed for the cells showed normal distribution of data for the red fluorescent cells (p > 0.05) and the Levene test revealed homogeneity of the variances for individual samples (p > 0.05). Statistically significant differences were demonstrated by one-way ANOVA with Tukey's HSD post-hoc test for significance levels (supplementary materials). One-way ANOVA for the red fluorescent cells showed values F (3.8) = 760.168; p = 0.00 (in the supplementary materials). The value of the coefficient ω^2^ = 99.53% indicated that the incubation with the successive concentrations of the protein-polysaccharide fraction had a strong effect on the presence of red fluorescent cells in the *C. albicans* culture. The results of the tests are presented in the supplementary materials (Fig. S1, Table S1).

Staining with a mixture of Hoechst and propidium iodide shows necrotic and apoptotic cells. Additionally, when this mixture was used, it was possible to observe mitochondrial DNA fluorescing green. Figure 2 presents *C. albicans* cells after staining with Hoechst and propidium iodide. The images A and B in Fig. 2a show the control culture, and C-J show cells after the treatment with AAF. The nuclear DNA glows blue. The cell walls are poorly visible. In pictures C and D, the blue, fluorescent surface of the nuclei is larger than in the control cells, and green glowing mitochondrial DNA is additionally visible. Images E and F show the migration of mitochondrial DNA to nuclear DNA, while images G and H show the fusion of mitochondrial DNA and nuclear DNA into one structure. Images I and J show cells with enlarged nuclei formed after the fusion of mitochondrial DNA with nuclear DNA. A characteristic feature is the very bright glow, which proves that the cells are undergoing the early apoptosis process. Thickened cell walls are also visible in cells with enlarged nuclei and glowing mitochondrial DNA. The cells glowing pink are necrotic cells. Images A and B in Fig. 2b show control culture cells where nuclear DNA glows blue and cells undergoing the apoptotic process with fragmented genetic material fluorescing intense light blue (marked by arrows), as shown in pictures C–J.

Staining with a mixture of Hoechst and propidium iodide dyes was performed in 3 replications and normal (blue fluorescence), apoptotic (blue-white fluorescence), and necrotic (red fluorescence) cells were counted. For each concentration, approximately 140 cells were counted in each repetition. The Shapiro–Wilk and Levene tests allowed determination of the normal distribution (p > 0.05) and the homogeneity of variance (p > 0.05) for individual samples, respectively. The one-way ANOVA test with the Tukey’s HSD post-hoc test showed significant differences between the analyzed means. One-way ANOVA showed values F (3.8) = 58.110; p < 0.001 for normal cells, F (3.8) = 20.3725; p < 0.001 for apoptotic cells, and F (3.8) = 102,822; p < 0.001 for necrotic cells (supplementary materials). Coefficient ω^2^ had values of ω^2^ = 93.45% for normal cells, ω^2^ = 82.89% for apoptotic cells, and ω^2^ = 96.22% for necrotic cells, which means that the protein-polysaccharide fraction in individual types of cells has a significant effect on each type of cells. The results of the tests are presented in the supplementary materials (Fig. S2, Tables S2 and S3).

**Figure 3:**
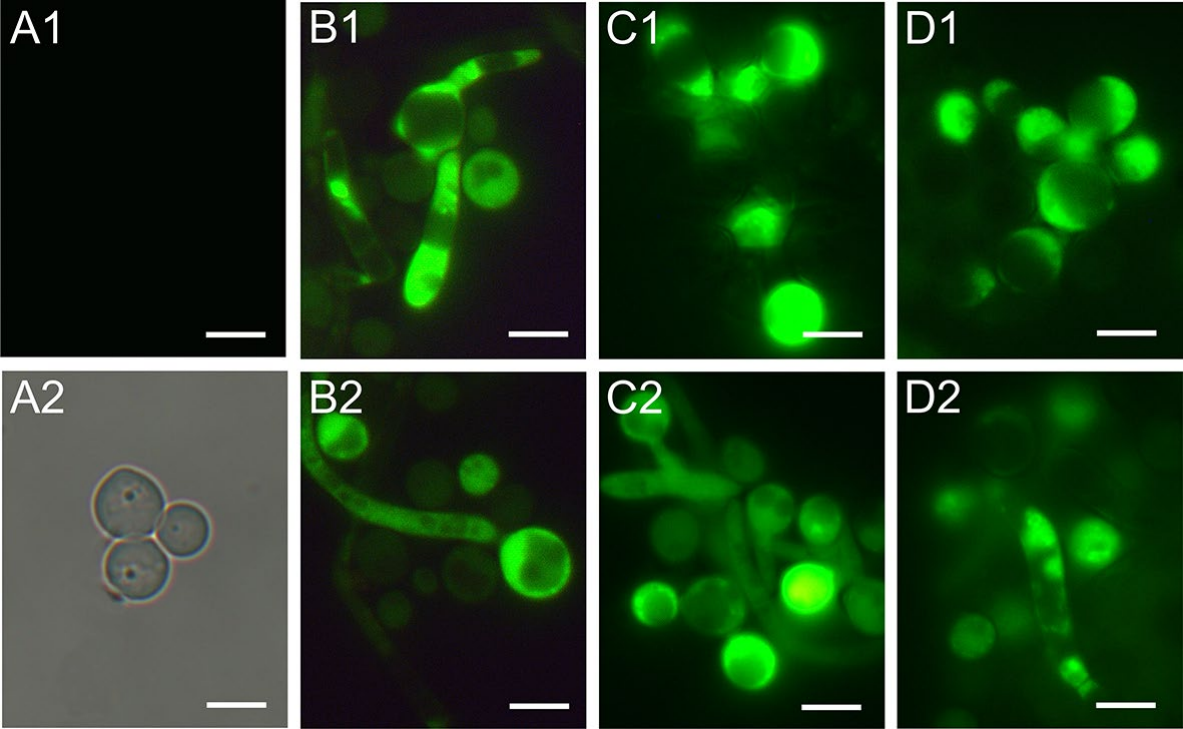
Detection of oxygen-reactive species in *C*. *albicans* cells from the: (**A1**,**A2**) control culture; A2 shows a bright field image of A1, and the AAF-treated cultures: (**B1**,**B2**) at the concentration of 25 µg mL^−1^, (**C1**,**C2**) 50 µg mL^−1^, and (**D1**,**D2**) 100 µg mL^−1^. The cultures were treated with AAF for 48 h. Fluorescence images were obtained after staining with 2′,7′-dichlorofluorescin diacetate (H_2_DCF-DA). Bars represent 5 µm.

Staining with 2′,7′-dichlorofluorescin diacetate (H_2_DCF-DA) is regarded as a rapid and sensitive assay for detection of reactive oxygen species (ROS) in response to oxidative stress in cells. The oxidized form of the dye (DCF) is highly fluorescent, and visible as green color localized in cytoplasm of the yeast cell. The representative results of oxidative stress in the *C. albicans* cells in response to the action of the AAF fraction are shown in Fig. 6. The presented effects were observed after 48 h of incubation of the fungal cells with the fraction; however, the first results were noticeable after an hour of the AAF action. The control *C. albicans* cells showed no fluorescence, which means that the polar form of H_2_DCF had not been oxidized inside the cells to the fluorescent form. Therefore, a bright field image (Fig. 3A2) was used to visualize the control cells. Picture 3 A1 is a fluorescent image of cells shown in picture 3 A2. After the action of the AAF fraction at each concentration, single cells or small groups of cells with clear green fluorescence located in the cytoplasm were visible. Strong fluorescence indicating intense oxidative stress was noticed both in the yeast cells and inside the hyphae and pseudohyphae. The intracellular fluorescence of the cytoplasm was clearly differentiating from the unstained vacuoles (Fig. 3B1,B2,C1,D1). The selected photos were representative of the 10 obtained images.

### Scanning and Transmission Electron Microscopy imaging of *C. albicans* cells after treatment with AAF

**Figure 4:**
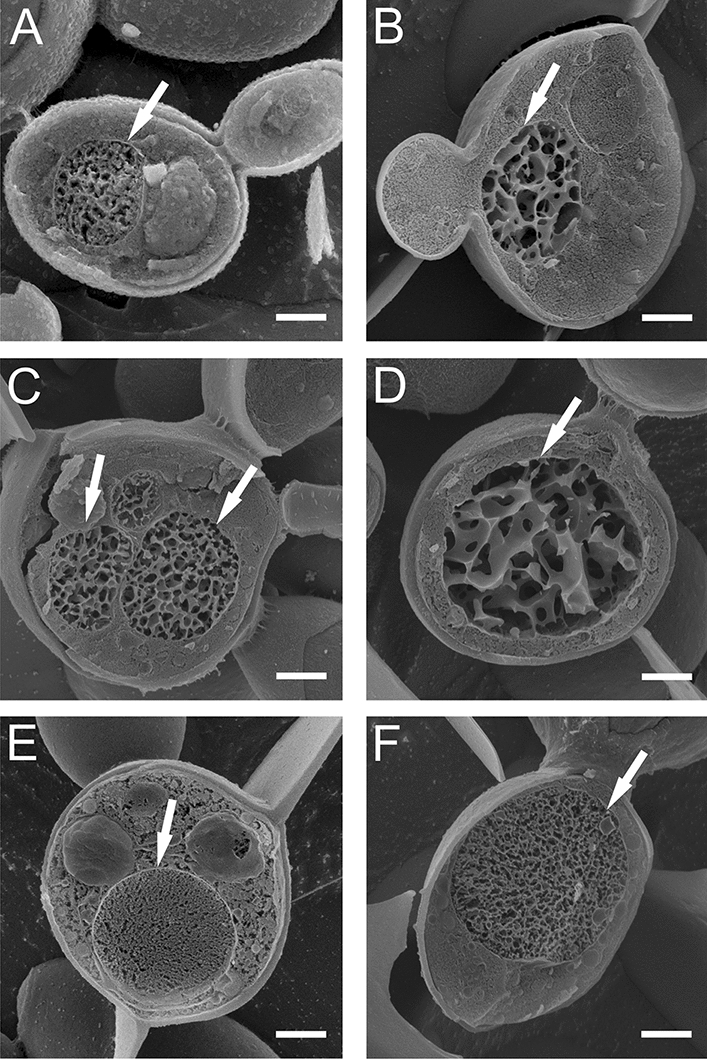
Cryo-SEM image of: (**A**) a control *C. albicans* cell; (**B**–**F**) *C. albicans* cells after incubation with AAF (100 µg mL^−1^); (**B**) a magnified cell with a proportionally enlarged nucleus, (**C**) a cell with three porous structures—two nuclei and a nucleolus, (**D**) a cell with a very large nucleus. E and F show cells with a clearly enlarged nucleus in comparison to the control. Bars represent 2 µm.

The Cryo-SEM method helps to visualize cell organelles without interference of fixatives in the cell structure. Using the Cryo-SEM technique, control cells and cells that retained cell wall integrity after the treatment with AAF (100 µg mL^−1^) were analyzed. Figure 4A shows a control culture cell with a clearly visible cell nucleus resembling a porous oval structure, marked with an arrow. The next images show the cell structure after the incubation with AAF. Image B presents a magnified cell with a proportionally enlarged nucleus, while image C shows a cell that contains three porous structures inside the cell—two nuclei and a nucleolus. In Fig. 4D, a cell with a very large nucleus filling almost the entire intracellular space is visible. Images E and F show cells with a clearly enlarged nucleus in comparison to that of the control cell. Each image is representative of the 10 obtained pictures.

**Figure 5:**
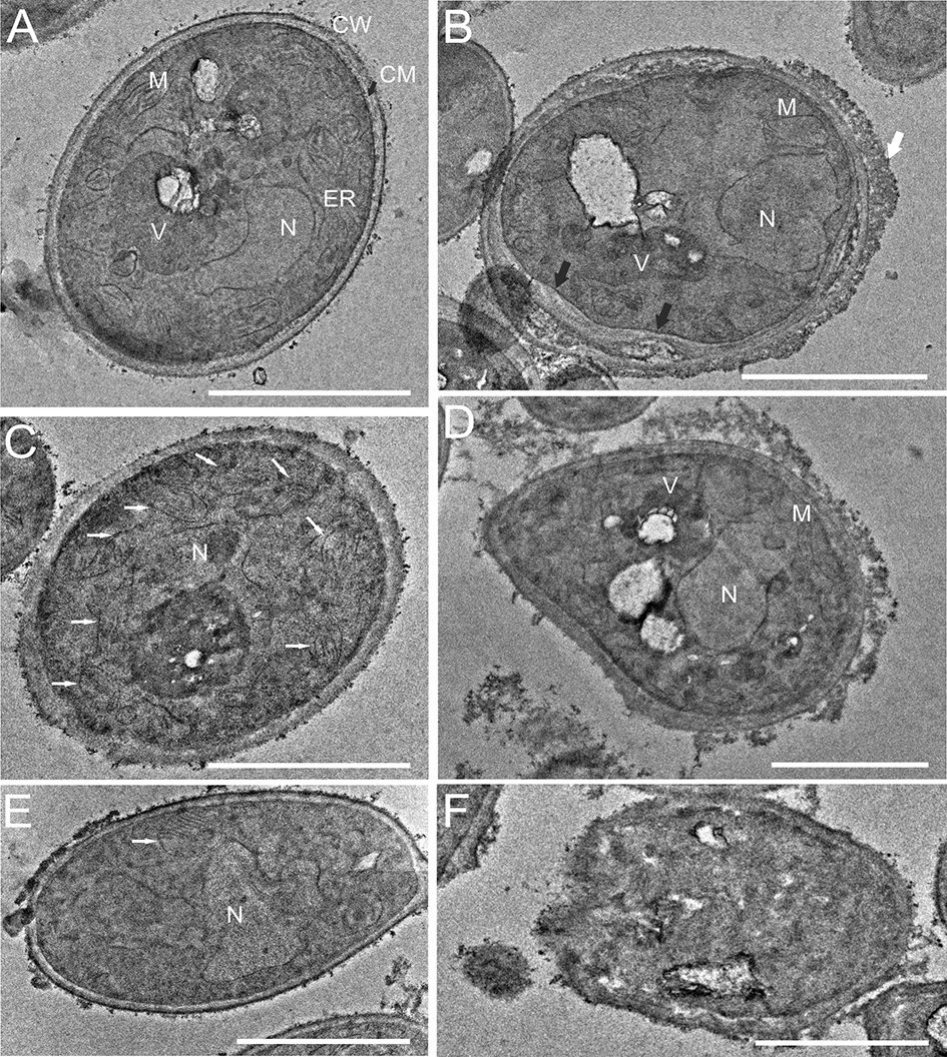
TEM image of (**A**) *C. albicans* control cell; *CW* cell wall, *CM* cytoplasmic membrane, *N* nucleus, *M* mitochondria; *ER* endoplasmic reticulum, *V* vacuole; (**B**–**F**) *C. albicans* cells after incubation with AAF (100 µg mL^−1^). (**B**) a cell with shrinkage of the cytoplasmic membrane (black arrows) and accumulated substance on the cell wall (white arrows); (**C**) a small cell with enlarged mitochondria (white arrows); (**D**) a deformed cell with fuzzy internal structures, (**E**) a cell with a large, scattered nucleus and a big mitochondrion marked by a white arrow, (**F**) a cell with loss of the shape of organelles and cell wall integrity. Bars represent 2 µm.

Transmission electron microscopy facilitates detailed observation of cellular structures that require a fixation process to maintain an unchanged form. The control cells were characterized by a smooth cell wall with regular thickness and clearly visible cell organelles (Fig. 5A). The correct morphology of the *C. albicans* cells can be compared with the image obtained by Nishiyama and collaborators^50^. After incubation with AAF, the fungal cells had an unevenly thickened wall on which a visible substance accumulated in the form of a mantle surrounding the cell from the outside, marked with white arrows in Fig. 5B. Shrinkage of the cytoplasmic membrane is indicated by black arrows in the same image. Image C shows a cell with enlarged mitochondria (marked by arrows) in comparison to the control cell. In picture D, the cell is clearly deformed and internal structures, excluding the nucleus, have fuzzy contours and highly diverse electron density. Picture E shows a cell with a large, diffused nucleus, and picture F presents a cell with loss of the shape of organelles and cell wall integrity. Each image is representative of the 10 obtained pictures.

**Figure 6:**
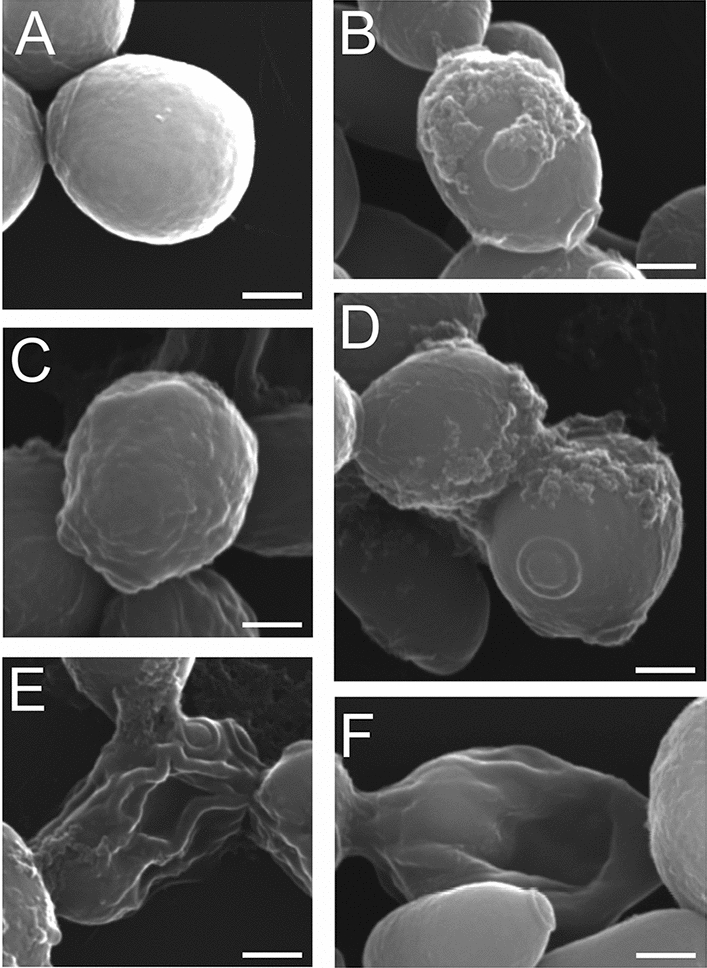
SEM images of: (**A**) *C. albicans* control cell, (**B**–**F**) *C. albicans* cells after incubation with AAF (100 µg mL^−1^). Images (**B**,**C**,**D**) cells with an irregular and rough surface; images (**E**,**F**) show collapsed cells. Bars represent 2 µm.

Scanning electron microscopy facilitates detailed observation of morphological changes in cells and on their surfaces. SEM analysis was performed on control culture cells and those treated with AAF at a concentration of 100 µg mL^−1^. In Fig. 6A, the control culture cells are visible as single cells with an oval shape and a smooth surface. Cells incubated with AAF exhibit visible division scars, and the coating substance is visible on their surface, as can be seen in Fig. 6B,F. In image C, the cell surface is irregular and rough. The cells shown in Fig. 6D,E are collapsed and not fully separated from each other. Each image is representative of the 20 obtained pictures.

### Identification of AAF components with the MED-FASP approach

To increase the number of protein identifications and the percentage of protein sequence coverage contained in the active fraction (AAF), we used the multi-enzyme digestion filter assisted sample preparation MED-FASP protocol^39^. Before digestion, water was added to the protein fraction and the concentration of the preparation was measured spectrophotometrically (2.3 mg mL^−1^). Precipitated forms were observed, and the preparation was vortexed and separated into two fractions by centrifugation and decantation. Lysis buffer containing both sodium dodecylsulfate and dithiotreitol was added to both fractions in accordance with the MED-FASP procedure. After heating to 95 °C, clear solutions were obtained in both cases. For further processing, 100 μg of protein was taken and digested on a 10 kDa membrane successively with LysC, trypsin, and finally chymotrypsin in agreement with the standard MED-FASP procedure^39^. After final C18 purification (see the “Materials and methods” section), each fraction was subjected to mass spectrometry analysis and protein identification. As a result, we obtained a list of 400 proteins (Table S4 in the supplementary materials). The main components of AAF, i.e., lysenin-related protein 2 (LRP-2) and lysenin, were identified with an accuracy of 95% and 90% sequence coverage, respectively (Fig. S3). According to their biological process in earthworms, they are involved in defense response to bacterium [GO: 0042742], hemolysis in other organisms [GO: 0044179], and ion transport [GO: 0006811]. In terms of molecular functions, they exhibit toxic activity [GO:0090729].

Other proteins from the lysenin group with importance for the biological activity of the preparation are lysenin-like proteins (fragment) with the sequence identified in 25% and lysenin-related proteins 1 and 3, where the amino acid sequence was confirmed in 16 and 38%.

The top twenty proteins also include proteins described as extracellular globins in our previous work^51^, based on the Uniprot database used for identification. They now function under the name erythrocruorins. These proteins are part of a large oxygen-carrying protein complex. Their molecular function is related to heme binding [GO: 0020037], metal ion binding [GO: 0046872], oxygen binding [GO: 0019825], and oxygen carrier activity [GO: 0005344]. We identified four such proteins (of the top twenty), and the Uniprot identifiers with the sequences are presented in Fig. S4. The sequence coverage in this case varies between 54 and 79%.

The entire analysis of biological processes, cellular components, and molecular functions based on the Uniprot data is presented in Table S5 in the supplementary materials. The first forty-two molecular functions (Table 1) indicate that most activities are associated with the broad-sense binding properties of AAF proteins. The binding involves components related to the genetic material (DNA and RNA binding), components used for the synthesis of nucleic acids (GTP binding), molecules responsible for cell energy (ATP binding), ion binding (e.g., iron, calcium, magnesium), heme binding, and oxygen binding and transfer.

**Table 1:** Molecular functions of proteins (with the number) identified in AAF.

GO therm with ID	Number of proteins in particular GO
ATP binding [GO:0005524]	65
Structural constituent of ribosome [GO:0003735]	36
GTP binding [GO:0005525]	35
Iron ion binding [GO:0005506]	34
GTPase activity [GO:0003924]	29
DNA binding [GO:0003677]	20
Protein heterodimerization activity [GO:0046982]	20
Metal ion binding [GO:0046872]	19
Calcium ion binding [GO:0005509]	16
Actin binding [GO:0003779]	15
Heme binding [GO:0020037]	14
Structural constituent of cytoskeleton [GO:0005200]	14
Actin filament binding [GO:0051015]	12
Magnesium ion binding [GO:0000287]	12
RNA binding [GO:0003723]	12
Oxygen carrier activity [GO:0005344]	11
Oxygen binding [GO:0019825]	10
Unfolded protein binding [GO:0051082]	10
ATPase activity [GO:0016887]	9
Manganese ion binding [GO:0030145]	8
Metalloaminopeptidase activity [GO:0070006]	5
NADP binding [GO:0050661]	5
Peptidyl-prolyl cis–trans isomerase activity [GO:0003755]	5
Translation elongation factor activity [GO:0003746]	5
Calmodulin binding [GO:0005516]	4
Catalase activity [GO:0004096]	4
Chromatin binding [GO:0003682]	4
Creatine kinase activity [GO:0004111]	4
fructose-bisphosphate aldolase activity [GO:0004332]	4
GTPase activator activity [GO:0005096]	4
NAD binding [GO:0051287]	4
Nucleic acid binding [GO:0003676]	4
Peroxidase activity [GO:0004601]	4
Protein serine/threonine phosphatase activity [GO:0004722]	4
Pyridoxal phosphate binding [GO:0030170]	4
Rab GDP-dissociation inhibitor activity [GO:0005093]	4
Toxin activity [GO:0090729]	4
Transferase activity [GO:0016740]	4
Ubiquitin conjugating enzyme activity [GO:0061631]	4

### Fishing in *Candida albicans* cell lysate pulled out many proteins associated with the cell surface

**Figure 7:**
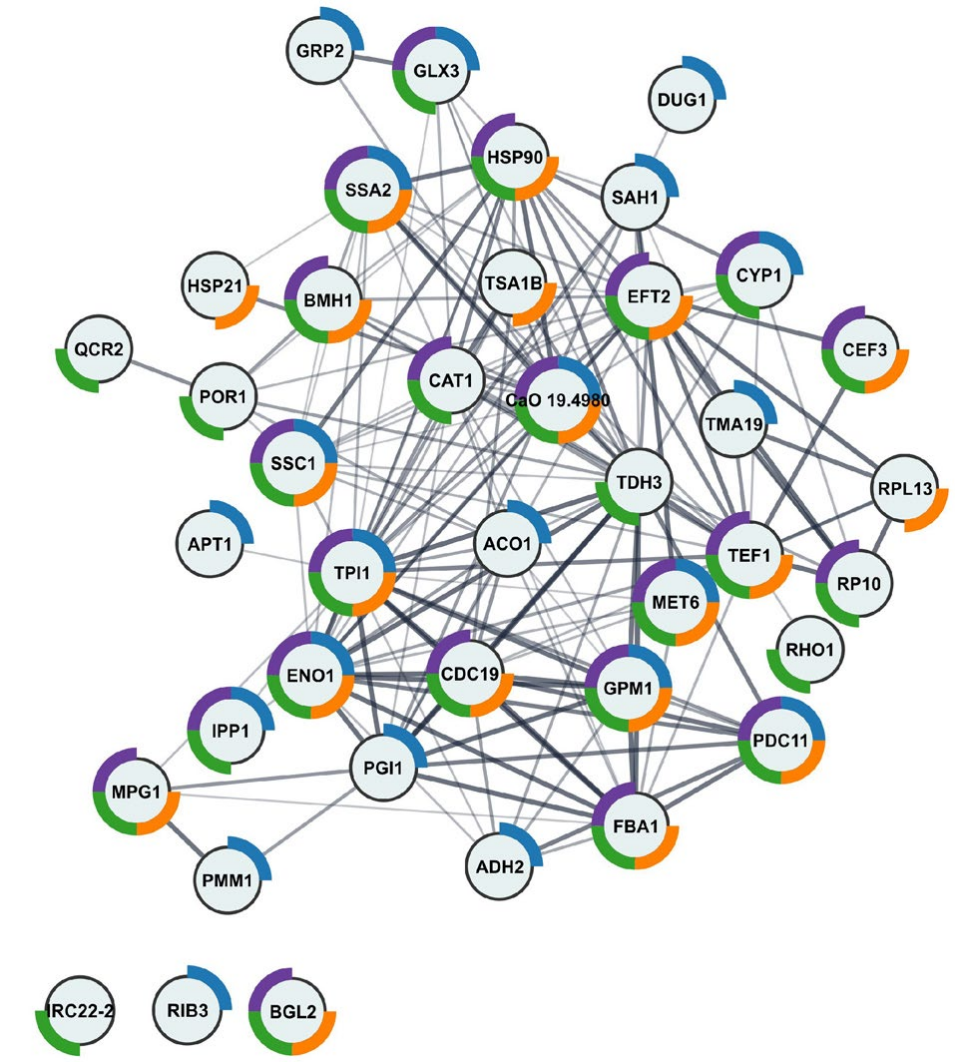
Graphical representation of the results of the functional analysis and the interaction network obtained using the STRING server for *Candida* proteins bound by the AAF fraction. The colors represent as follows: blue—fungal biofilm matrix, purple—fungal-type cell wall, green—cell periphery, orange—cell surface.

A simple affinity experiment was designed to test the interaction of AAF proteins with *Candida* proteins. The proteins from the preparation were deposited on a solid matrix that is commonly used for immobilizing proteins by amino groups. After preparing the small protein column on Cyanogen bromide-activated sepharose (CNBr), *C. albicans* cell lysate was loaded onto the column. After two hours of agitation, collection of unbound proteins, and washing the sepharose with clean buffer, the last milliliter of the wash was collected as a negative control before eluting proteins related to the deposited preparation. The dissociation of the complexes was carried out by adding acidic water (0.1% TFA in water). The elution fraction and the wash fraction were subjected to enzymatic digestion on the membrane following the FASP protocol. The recorded MS/MS spectra were used to identify proteins based on the *Candida* database (the Annelida database served as the basis for potential contaminants). The results of the protein identification in Peaks Studio software, biological processes, cellular components, molecular function, and pathways for all fished proteins are presented in Table S5 in the supplementary materials. The protein IDs were analyzed in the STRING and Cytoscape programs and the results are shown in Fig. 7. Sixty-five of the 96 identified proteins were implemented into the STRING server. Of these, 40 proteins in GO cellular components were shown to be interconnected, as visualized by the generated network from the STRING server and were directly related to the cell surface (Table S5). The presence of essential proteins representing heat shock proteins (HSPs), e.g., Hsp90, Hsp70, Hsp21, and Hsp60 as well as Ssa1 and Ssa2 was also shown. Table 2 lists all proteins retrieved from the lysate with the GO description, showing that proteins that were not qualified by the server functional analysis are also associated with maintenance of cell wall and cell membrane integrity. A large group of the proteins fished out comprises ribosomal units, both belonging to the ribosome structural constituent (Rpl) and the small cytosolic subunits of the ribosome (Rps).

**Table 2:** Proteins with statistically significant changes in concentrations in response to the presence of different concentrations of the AAF fraction with an over 1.5-fold change in at least one comparison.

Uniprot ID	Protein name	C25 to CK		C50 to CK		C100 to CK	
		p-value	Fold Change	p-value	Fold Change	p-value	Fold Change
G1UA77	Repressed by TUP1 protein 5	0.0002	3.13	6.38E−06	5.54	3.12E−05	7.87
C4YHL4	Superoxide dismutase [Cu–Zn]	1.68E−05	4.08	2.50E−06	4.13	5.44E−06	5.27
C4YCX4	Uncharacterized protein	0.00241	1.65	1.40E−04	3.26	0.00047	2.05
C5K446	High-affinity glucose transporter	0.43319	1.14	0.01.571	1.68	0.00995	1.76
A0A1D8PDU9	Glutamate synthase (NADH)	0.25717	1.13	0.0102	0.54	0.15724	1.07
B9WBH7	White colony protein, putative	0.00046	1.62	0.0023	1.22	0.43515	0.97
Q59X49	Stress protein DDR48	0.19949	0.95	0.0003	1.64	0.02674	0.87
A0A1D8PDX5	Isoleucine biosynthesis protein	0.02815	0.65	0.04272	0.78	0.00997	0.64
Q59U59	Proteinase A	0.89544	1.01	0.11298	0.95	0.02987	0.60
C4YM77	Uncharacterized protein	0.00021	0.62	0.00018	0.58	1.09E−05	0.53
Q59QN6	Formate dehydrogenase	0.00019	0.69	3.83E−05	0.48	2.59E−06	0.30
Q9P871	Copper transport protein 1	2.03E−05	0.30	5.77E−05	0.28	6.44E−06	0.10

### SWATH analysis of the influence of the AAF concentration on *C. albicans* cells

**Figure 8:**
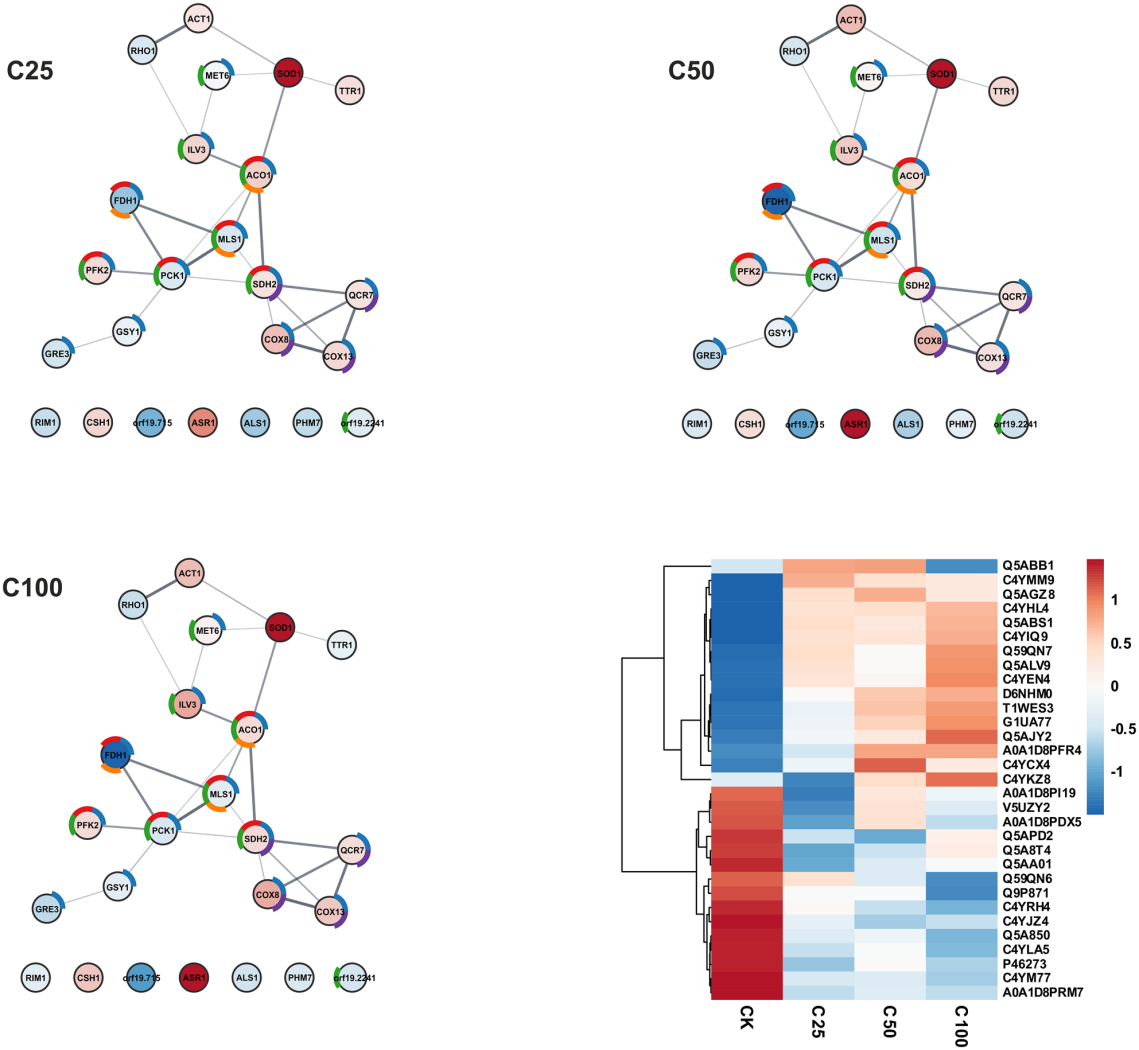
Graphical representation of the results of the quantitative functional analysis of the response of the *C. albicans* proteome to the AAF fraction. The color of the circles corresponds to fold changes: red for higher than 1, and blue for lower than 1. The color intensity indicates the size of the change. The donut chart colors correspond to KEGG pathways—blue: metabolic pathways, red: carbon metabolism, green: biosynthesis of secondary metabolites, orange: glyoxylate and dicarboxylate metabolism, purple: oxidative phosphorylation. The SWATH heatmap represents the comparison of the median of each sample with the t-test control sample (proteins with a p-value below 0.05 in each comparison).

The study of the effect of the AAF fraction and its concentration on the *Candida* cell proteome was carried out with the SWATH-MS methodology. After the treatment with three different AAF concentrations (25, 50, 100 µg mL^−1^), the yeast cells were collected, washed, and lyophilized. Thus, the preparations were subjected to cell lysis followed by digestion on a 10 kDa membrane (see the “Materials and Methods” section). Spectra recorded in the data-dependent acquisition (DDA) mode were used to prepare a SWATH library, which finally contained 1.353 proteins. Then, data-independent acquisition (DIA) spectra were recorded in triplicate for each sample and analyzed. Statistical analysis was carried out for the entire data package. Table S6 in the supplementary materials contains all the results obtained in the SWATH-MS measurements. We selected statistically significant proteins with a p-value below 0.05 for each AAF fraction concentration. Further functional enrichment analysis of interaction networks was performed in the STRING server and presented in a Fig. 8 and protein heatmap.

Significant upregulation was observed for essential in methionine synthesis cobalamin-independent methionine synthase (Met6) and Fe–S cluster enzyme, putative dihydroxy acid dehydratase (Ilv3) involved in the biosynthesis of branched amino acids such as valine, leucine and isoleucine and actin (Act1). Proteins associated with mitochondrial activity, such as aconitase hydratase (Aco1), components of the cytochrome c oxidase Cox13, Cox8, and Cox2, succinate dehydrogenase (Sdh) that is involved in complex II of the mitochondrial electron transport chain and is also upregulated responsible for transferring electrons from succinate to ubiquinone (coenzyme Q), cytochrome b-c1 complex subunit 7 (Qcr7). The most notable increases were observed for superoxide dismutase Sod1. In comparison to the untreated *C. albicans* cells, changes can be observed even at the lowest concentration of the preparation. At 25 µg mL^−1^, the fold change for Sod1 is 4.08, which increases by 4.13 to 5.27 as the concentration increases from 50 µg mL^−1^ to 100 µg mL^−1^. Another protein is the glycosylphosphatidylinositol (GPI)-anchored cell wall protein involved in utilization of hemin and hemoglobin for iron in the host. The Rbt5 fold change increases correspondingly with the increasing AAF concentration from the value of 3.13 through 5.54 to the value of 7.87. Rbt5 is not shown in the network presented in Fig. 8 due to the absence of the identifier (protein ID) of this protein in the STRING server database. The third protein showing a clear upregulation trend is the stress-responsive alcohol sensitive ring/PHD finger protein Asr1. The fold change is slightly different here, i.e., it is 1.65 at the lowest AAF concentration, increases to 3.226 at the AAF concentration of 50 µg mL^−1^, and drops to 2.05 at the highest value of 100 µg mL^−1^. In addition to Asr1, there is another upregulated protein outside the STRING net (Fig. 8), i.e., aldo–keto reductase Csh1.

In the case of downregulated proteins, the most evident fold change caused by the exposure to AAF is evident for one protein, i.e., formate dehydrogenase Fdh1, which catalyses the NAD^+^- dependent oxidation of formate to carbon dioxide. As the AAF concentration increases, the fold change value drops to 0.3. Other proteins whose concentration is changed by the presence of even the lowest AAF concentration are two proteins connected with cell wall, namely GTP-binding protein Rho1 regarded as a predominant regulatory component of glucan synthase and cell surface adhesin Asl1. Glutathione reductase Ttr1, also known as Grx2 in *S. cerevisiae*, malate synthase Mls1 from the glyoxylate cycle, glucose repressible phosphoenolpyruvate carboxykinase Pck1, glycogen synthase Gsy1, and aldose reductase Gre3 are other downregulated proteins. Besides the STRING network (Fig. 8), the downregulated proteins are represented by the mitochondrial single-stranded DNA binding protein Rim1 involved in phosphate metabolism Phm7, the unknown protein Orf19.715, and the Orf19.2241 Pst1p protein, which displays typical features of GPI-anchored proteins.

### XPS analysis of *C. albicans* after treatment with AAF

**Figure 9:**
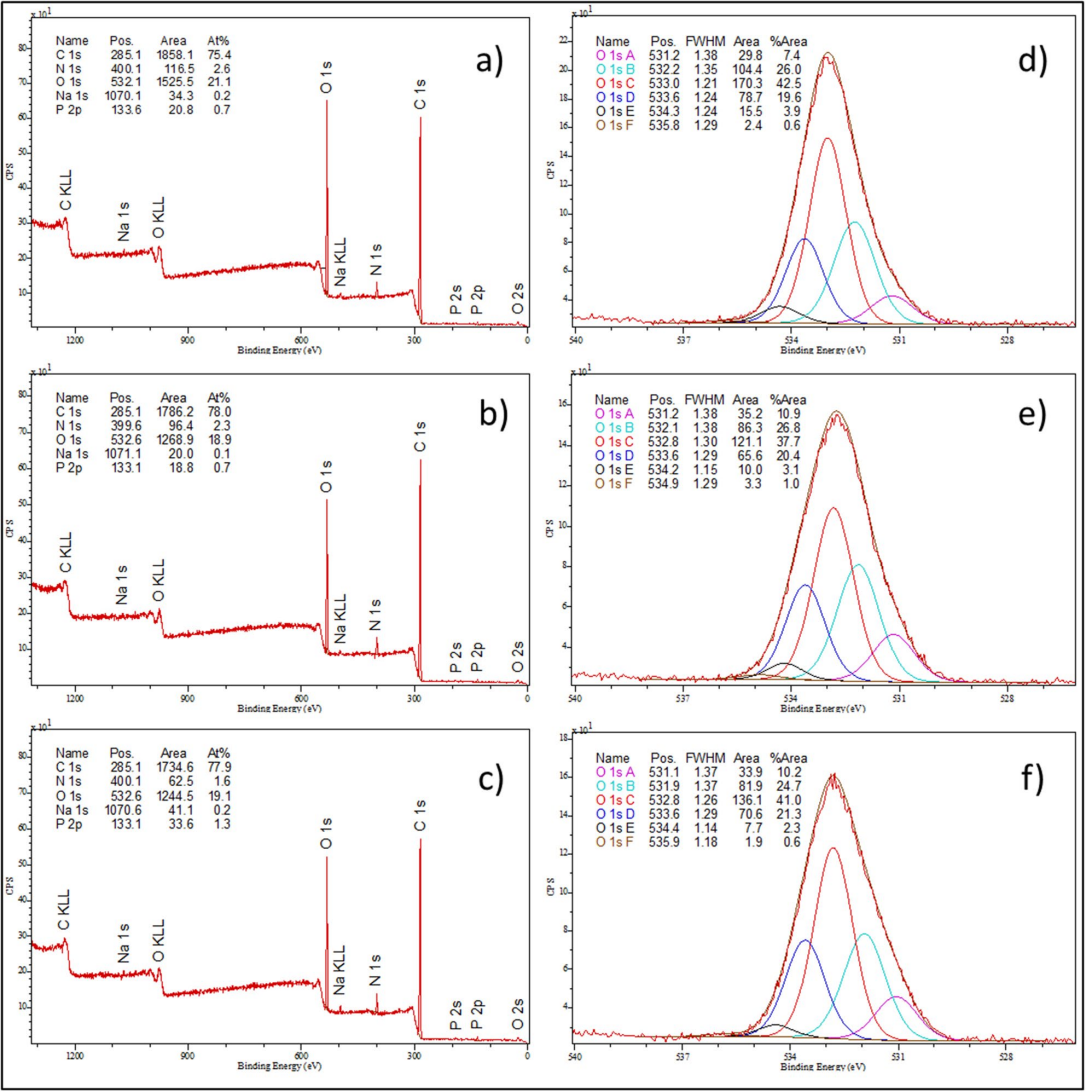
XPS spectra over a wide range of binding energies: (**a**) *C. albicans* control cells, (**b**) *C. albicans* cells incubated with AAF at a concentration of 50 µg mL^−1^, (**c**) *C. albicans* cells incubated with AAF at 100 µg mL^−1^; XPS spectra in a narrow range of binding energies characteristic for oxygen: (**d**) *C. albicans* control cells*,* (**e**) *C. albicans* cells incubated with AAF at a concentration of 50 µg mL^−1^, (**f**) *C. albicans* cells incubated with AAF at 100 µg mL^−1^.

XPS analysis was used to analyze the elemental composition of the surface of the tested cells. To determine the elemental composition of the *C. albicans* control cells and those incubated with AAF at a concentration of 50 µg mL^−1^ and 100 µg mL^−1^, the XPS spectrum was obtained from the surface of the samples in a wide range of binding energy (Fig. 9a-c). The results of the elemental composition analysis are presented in Table 3^52–54^.

**Table 3:** Quantitative results of the XPS elemental composition analysis of the surface of *C. albicans* control cells and *C. albicans* cells incubated with AAF at a concentration of 50 µg mL^−1^ and 100 µg mL^−1^.

Sample identifier	Name	Position	Raw Area	%At Conc	% St.Dev
*C. albicans* control cells	C 1s	285.1	1931.200	75.1	2.06
	N 1s	400.1	123.421	2.7	1.81
	O 1s	532.6	1611.350	21.4	1.53
	Na 1s	1070.1	34.478	0.2	0.25
	P 2p	133.6	21.741	0.7	0.60
*C. albicans* treated with AAF at 50 µg mL^−1^	C 1s	285.1	1828.060	77.0	2.72
	N 1s	399.6	122.229	2.9	2.62
	O 1s	532.6	1345.800	19.4	1.73
	Na 1s	1071.1	22.673	0.1	0.24
	P 2p	133.1	19.314	0.7	0.95
*C. albicans* treated with AAF at 100 µg mL^−1^	C 1s	285.1	1778.820	76.6	2.96
	N 1s	400.1	109.758	2.6	2.96
	O 1s	532.6	1319.420	19.4	1.84
	Na 1s	1070.6	41.169	0.2	0.24
	P 2p	133.1	33.602	1.2	0.92

The XPS tests showed the following elemental composition of the *C. albicans* control sample: carbon—75.1%, nitrogen—2.7%, oxygen—21.4%, sodium—0.2%, and phosphorus—0.7%. The analyses showed the greatest changes in the oxygen concentration in the *C. albicans* samples caused by the exposure to 50 µg mL^−1^ and 100 µg mL^−1^ of AAF. The incubation with AAF decreased the oxygen content from 21.4% to 19.4%, which is a significant change in the case of these analyses. The fluctuations in the oxygen concentration may have been caused by oxidative stress related to the formation of free radicals damaging the protein and DNA in the *C. albicans* cells.

The XPS tests were also performed in the narrow binding energy range characteristic for oxygen. The results are presented in Fig. 9d–f and in Table 4.

**Table 4:** Quantitative results of the XPS analysis of the elemental composition of the surface of *C. albicans* control cells and *C. albicans* cells incubated with AAF at the concentration of 50 µg mL^−1^ and 100 µg mL^−1^.

Sample identifier	Name	Position	Raw area	%At Conc	Groups
*C. albicans* control cells	O 1s A	531.20	29.817	7.4	O^−2^
	O 1s B	532.24	104.367	26.0	O=C
	O 1s C	532.99	170.318	42.5	C–OH
	O 1s D	533.63	78.746	19.6	C–O–C
	O 1s E	534.31	15.463	3.9	O=C–O–
	O 1s F	535.78	2.414	0.6	H_2_O/O_2_
*C. albicans* treated with AAF at 50 µg mL^−1^	O 1s A	531.16	35.179	10.9	O^−2^
	O 1s B	532.14	86.304	26.8	O=C
	O 1s C	532.82	121.130	37.7	C–OH
	O 1s D	533.61	65.584	20.4	C–O–C
	O 1s E	534.19	10.041	3.1	O=C–O–
	O 1s F	534.89	3.301	1.0	H_2_O/O_2_
*C. albicans* treated with AAF at 100 µg mL^−1^	O 1s A	531.05	33.875	10.2	O^−2^
	O 1s B	531.94	81.947	24.7	O=C
	O 1s C	532.80	136.062	41.0	C–OH
	O 1s D	533.59	70.592	21.3	C–O–C
	O 1s E	534.41	7.660	2.3	O=C–O–
	O 1s F	535.89	1.889	0.6	H_2_O/O_2_

The XPS tests contributed to identification of the chemical bonds of oxygen present in the tested samples. The incubation of *C. albicans* with AAF caused the greatest changes within the O^2−^ groups. After the incubation, a significant increase in oxygen bonding in the O^2−^form was observed. An increase in the number of C–O–C bonds was also observed along with the increase in the AAF concentration.

### Spectroscopic analysis of AAF

Energy dispersive X-ray *spectroscopy* (*EDS*) facilitates identification of the elemental composition present in the analyzed sample. EDS analyses were performed for five selected areas, and the arithmetic mean was calculated. The research showed the following elemental composition of the preparation: carbon—58.67%, nitrogen—14.51%, oxygen—23.70%, sodium—1.84%, magnesium—0.02%, phosphorus—0.04%, sulfur—0.24%, chlorine—0.85%, potassium—0.04%, and calcium—0.08%. Moreover, the presence of iron, copper, and zinc in an amount not exceeding 0.01% was found in some of the analyzed areas (Table 5). These analyses confirmed the high homogeneity of AAF.

**Table 5:** Elemental composition of AAF.

Element	S1	S2	S3	S4	S5	Standard deviation
C	58.35	58.28	58.77	58.65	59.29	± 0.40
N	14.54	14.00	14.13	15.04	14.85	± 0.45
O	23.31	24.59	24.22	23.77	22.60	± 0.78
Na	2.07	1.91	1.83	1.60	1.79	± 0.17
Mg	0.04	0.02	0.02	0.02	0.01	± 0.01
P	0.05	0.04	0.03	0.03	0.04	± 0.01
S	0.27	0.24	0.22	0.22	0.25	± 0.02
Cl	1.23	0.79	0.66	0.56	1.00	± 0.27
K	0.05	0.03	0.03	0.03	0.05	± 0.01
Ca	0.08	0.07	0.08	0.06	0.09	± 0.01
Fe	–	0.02	–	0.01	0.01	± 0.01
Cu	–	–	–	0.01	0.01	± 0.00
Zn	–	–	0.01	0.01	0.01	± 0.00

**Figure 10:**
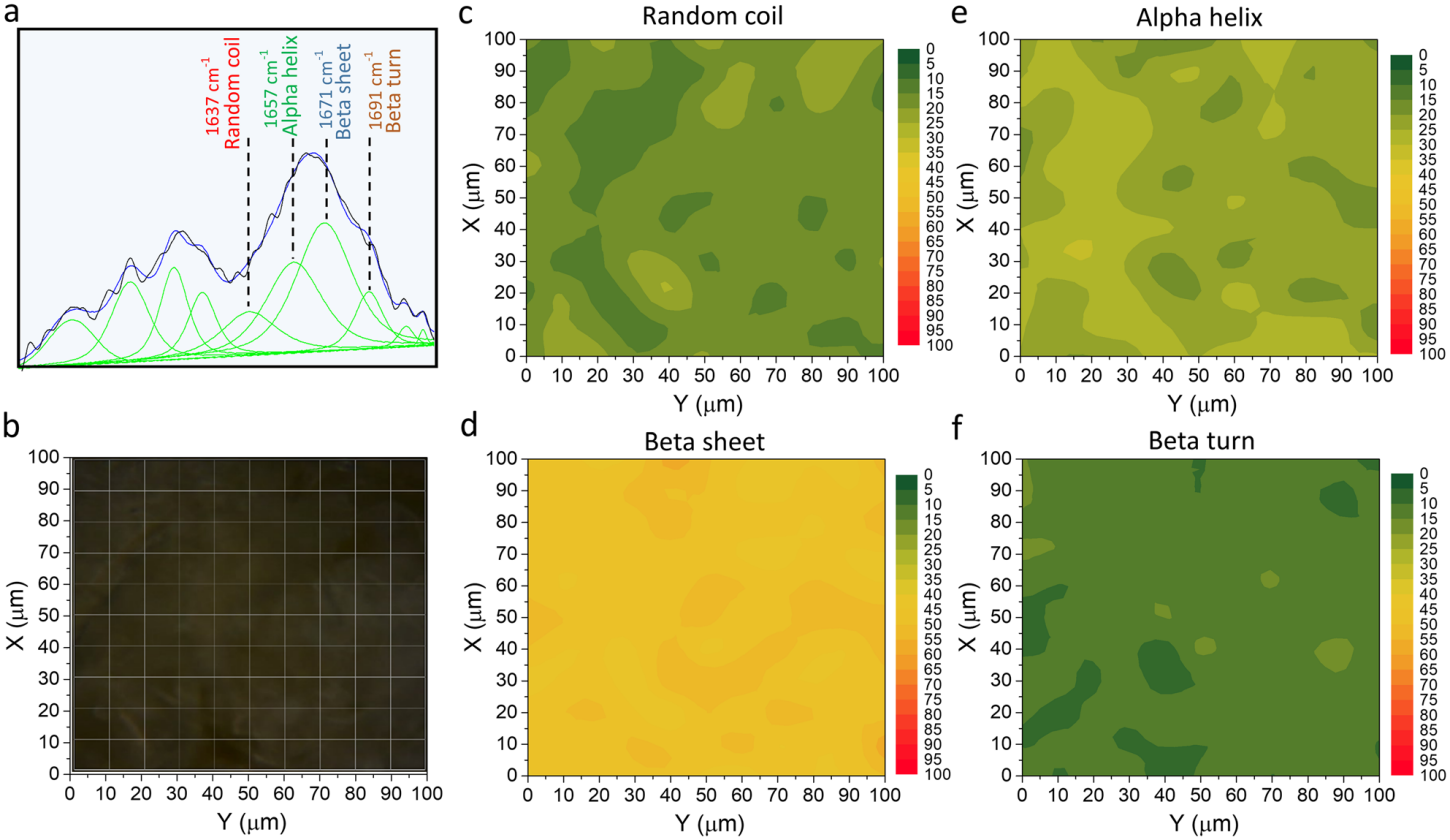
Raman spectrum of AAF with visible deconvoluted curves in the range from 1530 cm^−1^ to 1710 cm^−1^ (**a**) photograph of the analyzed AAF surface with the grid of Raman spectra measurements; (**b**) Raman maps presenting the percentage distribution of the random coil; (**c**) beta sheet (**d**) alpha helix (**e**) and beta turn (**f**) structures on the AAF surface.

Raman spectra mapping was effectively used to determine the distribution of the secondary structure of proteins in our earlier research^55,56^. The distribution of the protein secondary structure on the AAF surface is presented in Fig. 10. Figure 10a shows the curve-fitting process in the spectral range from 1530 cm^−1^ to 1710 cm^−1^. This range comprises the Amide I band, which was used to estimate the percentage content of individual secondary structures. Bands assigned to the alpha helix, beta sheet, beta turn, and random coil structure are labeled in Fig. 10a. The grid of the Raman spectra measurements with a photograph of the analyzed AAF surface is presented in Fig. 10b. The Raman maps in Fig. 10c–f show the percentage change in the protein content in the random coil, beta sheet, alpha helix, and beta turn conformation. The colors in the maps correspond to the percentage content of the secondary structure. Based on the maps, it can be concluded that the beta sheet and beta turn content changes from 40 to 58% and from 7 to 17%, respectively. In turn, the alpha helix and random coil content changes from 15 to 30% and from 12 to 26%, respectively. The average values of the beta sheet, beta turn, alpha helix, and random coil content are 47.7 ± 3.5%, 11.9 ± 2.1%, 23.3 ± 3.5%, and 17.0 ± 2.9%, respectively. These results are convergent with those obtained in our previous studies^30^.

The mass spectroscopy analysis shows that the main components of AAF are lysenin-related protein 2 (LRP-2) and lysenin. According to their biological process in earthworms, lysenin is involved in defense response to bacteria [GO: 0042742], hemolysis in other organisms [GO: 0044179], and ion transport [GO: 0006811]. In terms of molecular functions, they exhibit toxic activity [GO:0090729]. Hereć et al. showed that more than half of the residues in lysenin tend to form beta structures^57^. The percentage content of the secondary structure of proteins obtained from the analysis of Raman spectra indicates that beta structures account for about 58%. This result agrees with the mass spectrometry analysis results.

## Discussion

Previous studies have shown that the fraction of *D. veneta* coelomic fluid is active against various strains of *C. albicans*, i.e., a clinical strain and ATCC 10231, *C. krusei*, and ATCC 6258^30^. AAF was shown to be effective in reducing the metabolic activity of these strains. A clinical strain was selected for further detailed analysis of the effect of AAF on yeast cells. Incubation in the presence of AAF was shown to alter the cell wall, which consequently lost its integrity^51^. The *C. albicans* cell wall is responsible for cell integrity and interaction with the environment and is considered a pathogen-specific antifungal drug target^58,59^. Cell wall synthesis and remodeling is a dynamic process responding to growth conditions, cell cycle progression, changes in cell morphology, and cell wall stress. Defects in the integrity of the *C. albicans* cell wall have been shown to be functionally related to defects in mitochondrial morphology and phospholipid homeostasis^60^.

Our research was aimed at determination whether the changes in the cell wall were correlated with mitochondrial and nuclear processes. In previous studies, it was observed that multicellular forms appeared after AAF incubation^51^. Probably, AAF disrupts the normal coordination of membrane and nuclear events, leading to a state where the chromosomes that should separate are left without separation. It can be assumed that the dedifferentiation is accompanied by incorrect synthesis, replication, and distribution of genetic material to daughter cells. The observed SEM images indicate a profound disturbance in the cell cycle. It is known from the literature that, during *C. albicans* growth under stress, unequal DNA segregation leads to the formation of aneuploids. This process occurs in response to the action of antifungal azoles^61^.

Staining of cells with acridine orange indicates disturbances in metabolic processes in the cell. This cationic fluorochrome can cross the cell membrane and intercalate with DNA and RNA, which allows differentiation of cellular compartments with different pH^47,62,63^. Acidic cellular compartments glowed orange in many cells after the treatment with AAF. There were visible cells where the red–orange color spread throughout the cytoplasm, and the cells lost their natural shape and were clearly deformed. This was related only to non-viable cells. However, the percentage of metabolically inactive cells was significantly higher than just non-viable cells, as shown in previous studies (after using a concentration of 100 µg mL^−1^, the metabolic activity of *C. albicans* was reduced by as much as 90%)^30^.

It was previously observed that *C. albicans* cells underwent both necrosis and apoptosis after incubation with AAF^30^, but only now have the mitochondrial-nuclear phenomena preceding programmed cell death been noticed. Mitochondria are cellular organelles that produce metabolic intermediates used for the biosynthesis of amino acids and lipids, and mitochondrial respiratory chains are centers of energy production. Mitochondrial aerobic respiration plays a major role in *C. albicans* metabolism. The cells of this fungus need an energy supply and essential metabolites to survive, develop, and transform into hyphae. The images showing the early stage of apoptosis of *C. albicans* cells show blue-stained nuclear material and green-stained mitochondrial DNA. It is very clear that the mitochondrial DNA gradually moves towards the nuclear DNA and then these structures are joined. The effect observed using the fluorescence microscope is confirmed by the images obtained with the Cryo-SEM technique, where two porous structures corresponding to the image of the nucleus are visible, and one large structure probably formed after the fusion of mitochondrial and nuclear DNA. The mechanism of the escape of mitochondrial DNA to the nucleus is known from the literature and has been described in the yeast *Saccharomyces cerevisiae*^64–66^. The phenomenon of migration of fragmented mitochondrial DNA (mtDNA) to the nucleus has been proven and described in many organisms, including yeast, plants, and mammals^66^. The studies conducted so far suggest that mtDNA transfer to the nucleus is still an evolutionary process leading to de novo disruption of nuclear genes^67–70^. Studies on yeast as a model organism have revealed that fragmented mtDNA is captured during the repair of induced double-stranded DNA breaks in nuclear chromosomal or plasmid DNA^71–73^.

The appearance of large vacuoles in cells^51^ allows putting forward a hypothesis of a phenomenon of vacuolar d0egradation of mitochondria in yeast, which is one of the pathways of mtDNA escape to the nucleus^62^. Changing the conditions of growth, in our case the enrichment of the nutrient medium with AAF, may initiate the reprogramming of cellular metabolism via turnover of a portion of the mitochondrial complement. Cells containing damaged mitochondrial compartments tend to remove these organelles selectively, as defective mitochondrial compartments may alter cellular metabolic activities. The process of removal of those compartments via a salvage pathway would help optimize the metabolism in the cell. In addition, it is important that damaged mitochondrial compartments are more likely to generate reactive oxygen species that can damage DNA, lipids, and proteins^74^. There is ample evidence proving that, in higher eukaryotes, damaged mitochondrial compartments in a susceptible cell can inappropriately trigger cell death through the untimely loss of cytochrome c, i.e., a positive effector of apoptosis^75–77^. This fact may explain the confirmed activity of AAF on lung cancer cells A549^31^ and human colon adenocarcinoma cells^32^.

The proteome response of *C. albicans* cells to the presence and increasing concentrations of AAF was investigated by mass spectrometry and label-free quantification according to the SWATH methodology. The *C. albicans* cells were treated with three different concentrations of AAF. The number of individual proteins in the response to AAF was compared with that in control cells cultured in identical conditions as the AAF-treated cells. The analysis showed a clear response of the *Candida* proteome even at the lowest concentration (25 µg mL^−1^ AAF). The selected proteins with statistically significant changes in the presence of the lowest concentration maintain a change tendency across all the given concentrations. Significant overexpression of the Rbt5 and Sod1 proteins appeared already at the lowest AAF concentration, and the overexpression effect increased with the increasing dose of the preparation.

One of the main factors of microbial virulence is the ability to extract iron from host tissues. *C. albicans* can use hemin and hemoglobin as a source of iron, but the molecular basis of this process is not fully understood. The mannosylated protein Rbt5 is strongly induced by iron starvation^78^. Superoxide dismutases (SODs) are enzymes catalyzing the conversion of superoxides to hydrogen peroxide and molecular oxygen. There are two common types of enzymes in eukaryotic cells: cytoplasmic Cu/Zn superoxide dismutase and mitochondrial matrix-localized Mn superoxide dismutase^79^. It has been proved that superoxide dismutases in *Saccharomyces cerevisiae* are encoded by two genes: cytoplasmic SOD by Sod1 and mitochondrial SOD by Sod2^80,81^. The essential role of SOD is to protect cells against various types of stress^82,83^. Cytoplasmic superoxide dismutase is involved in the overall antioxidant capacity of the cell to a greater extent than the mitochondrial isoform^84,85^.

Only Asr1 exhibited an increase at 50 µg mL^−1^ AAF and a decrease at 100 µg mL^−1^, being greater than the highest dose used. Regardless of the concentration of the preparation applied, the expression of the other upregulated proteins was similar as in the control cells. In the case of downregulated proteins, only the expression of Fdh1 seemed to be sensitive to the changes in the concentration of substances present in the AAF fraction. It was evident that its amount decreased with the increase in the AAF concentration. The other proteins from this group did not show significant changes in expression under the influence of the increasing AAF concentration.

Analyzing the functions of proteins, the expression of which reflects the presence of substances contained in the active fraction of the AAF, it can be seen that the cells react by decreasing concentrations of the key proteins which activity is associated with cell growth and fermentation: for carbohydrate biosynthesis like Mls1 (glyoxylate cycle)^86^ Pck1 in the case of gluconeogenesis^87^, glycan biosynthesis in the case of Gsy1^88^ and Fdh1 which participate in methanol oxidation path^89^. Cells increase the level of enzymes involved in the synthesis of methionine (Met6)^90^ and branched chain amino acids (Ilv3)^91^. The effect of the tested AAF fraction on *C. albicans* cells begins with the negative influence and partial destruction of the cell wall, which confirms the response of the surface proteome. Cells in contact with the earthworm proteins begin to interfere with the cell wall architecture and overproduce the protein anchored to the membrane and responsible for hem-iron utilization—Rbt5^78,92^. This protein exhibited the greatest response to the presence of the AAF fraction, with a fold change up to 7.89 in the case of the 100 µg mL^−1^ AAF concentration. The protein is important for the morphogenesis of the *C. albicans* cell wall, and the highest fold change value in contact with the anti-candidal agents from the AAF preparation suggests that it is a necessary protein for *Candida* in these conditions. The cell tries to maintain cell wall morphogenesis and the activity of other proteins related to the biosynthesis of wall components. Another important process that affects the action of AAF on *Candida* cells is the presence of ROS (Reactive Oxygen Species) generated by aerobic respiration and oxidation of substrates.

Free radicals are molecules that contain at least one unpaired electron on the outer electron shell. They are highly reactive, as they seek to pair electrons by taking them away or giving them away to other molecules. In living organisms, free radicals are formed e.g., because of the impact of physical factors on the cell (e.g., ultraviolet radiation, ionizing radiation, ultrasound, or increased temperature) and through the metabolism of various exogenous chemical compounds, including drugs, in the proper course of many life processes. They play a role in the regulation of gene expression, protein phosphorylation processes, or calcium concentration in cells. They also activate proteins that control cell division and participate in elimination of microorganisms. However, the excess of free radicals leads to the destruction of structural and functional elements of cells, disturbances in homeostasis, and apoptotic or necrotic death^93,94^.

The response allowing regulation of the level of toxic forms involves an increase in the activity of proteins involved in defense, in this case superoxide dismutase Sod1^95,96^. In the present experiments, the cells reacted already at the 25 µg mL^−1^ concentration of the preparation by increasing Sod1 activity, which was enhanced with the increasing concentration. This suggests that contact with the compounds contained in the AAF fraction causes oxidative stress, which the cell compensates for by triggering ROS-removing superoxide dismutase 1 to keep the cell alive. Such a cellular response has already been described for other compounds with anti-*Candida* properties, such as the *C. albicans* response to the presence of sophorolipid^97^. Mitochondria, which are associated with respiration and aerobic metabolism, are the main source of ROS in the cell. Proteomic functional analysis has shown upregulation in the case of proteins that are closely related to mitochondrial activity: Aco1, Sdh2, Qcr7, Cox2, Cox8, and Cox13^98^. This activation remains constant regardless of the AAF concentration used.

Many natural compounds with anti-*Candida* activity targeting the mitochondria of these cells have been described. For example, berberine from *Berberis vulgaris* herb shows antifungal activity by inducing mitochondrial dysfunction and increased generation of ROS. Furthermore, the use of berberine disrupts the integrity of the *C. albicans* cell wall^99^. The synergistic action with fluconazole gives good results even in the case of fluconazole-resistant clinical isolates^100–102^. (+)-Medioresinol from the anti-inflammatory and analgesic *Sambucus williamsii* herb, used against *C. albicans*, can induce ROS production, and induce apoptosis following cell cycle arrest^99,103^. Garlic allyl alcohol (*Allium sativum*) has antifungal activity by inducing oxidative stress, such as increasing the reduction of ROS and depleting glutathione^104^. Baicalin can inhibit the activity of mitochondrial enzymes (such as Ca^2+^ -Mg^2+^ -ATPase, succinate dehydrogenase, and cytochrome oxidase) and induce cell cycle blockade and apoptosis^105^. Curcumin, i.e., the yellow pigment isolated from plant *Curcuma longa*, may increase ROS production and apoptosis in *C. albicans* cells and has synergistic effects with anti-fungal drugs such as azoles and polyenes^106,107^. Shikonin isolated from *Lithospermum erythrorhizon* can induce the endogenous ROS production, reduce the mitochondrial membrane potential, and have an influence on mitochondrial aerobic aspiration^108^.

Research on the anti-*Candida albicans* effect of the analyzed preparation has application potential and therefore the invention entitled: “The high molecular weight fraction of the celomic fluid from the worm *Dendrobaena veneta* for the treatment of mycoses caused by *Candida albicans*” has been covered by patent protection by the Polish Patent Office under the number PL. 234801^109^. It is worth adding that most of these preparations, in addition to their antifungal activity, also exhibit antitumor activity^110,111^, which is a similar phenomenon to that observed in the analysis of the AAF activity. Although we previously recognized that the target of AAF is the cell wall, mitochondrial processes are closely related to loss of cell wall integrity, and mitochondria should be considered a very important target for antifungal therapy.

## Supplementary Information

Supplementary Figure 1.

Supplementary Figure 2.

Supplementary Figure 3.

Supplementary Figure 4.

Supplementary Information 5.

Supplementary Tables S1–S3.

Supplementary Table S4.

Supplementary Table S5.

Supplementary Table S6.
