# Supplementary figures and images for: Metabolic, structural, and proteomic changes in Candida albicans cells induced by the protein-carbohydrate fraction of Dendrobaena veneta coelomic fluid
Source: Sci Rep. 2021 Aug 18;11:16711. doi: 10.1038/s41598-021-96093-1 (PMC8373886; doi:10.1038/s41598-021-96093-1)

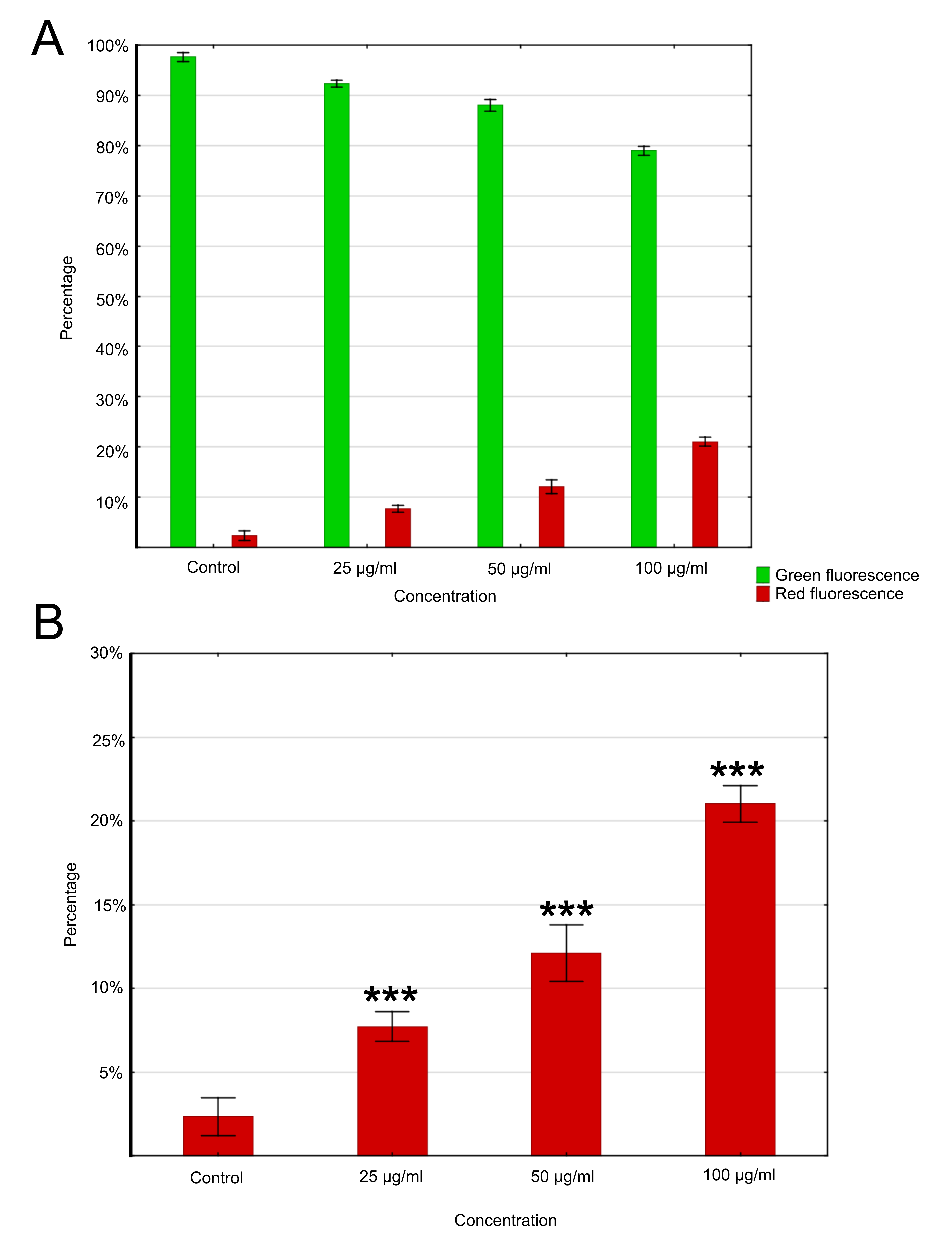

Supplement: Supplementary file 1 — Supplementary Figure 1. [file 41598_2021_96093_MOESM1_ESM.tif]

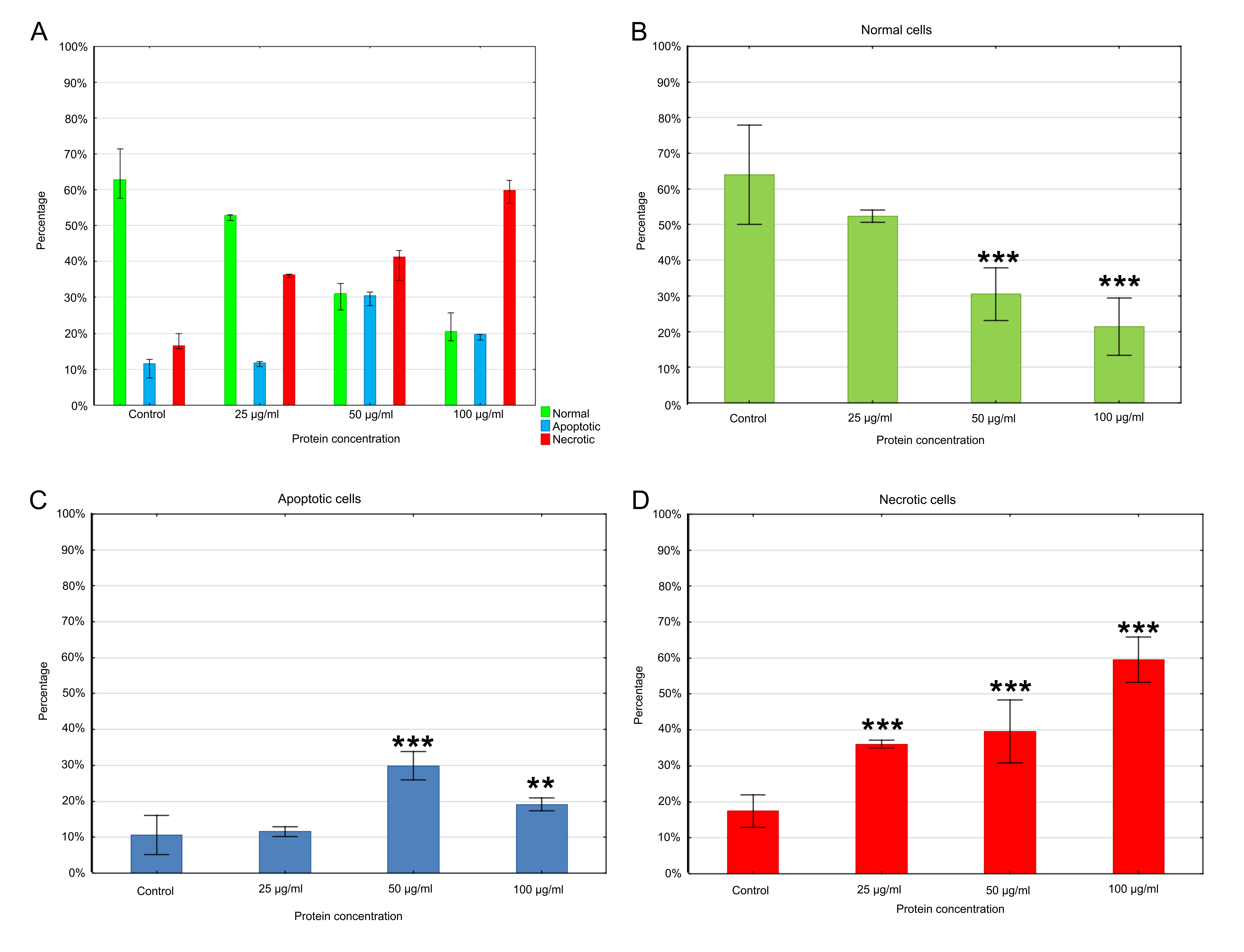

Supplement: Supplementary file 2 — Supplementary Figure 2. [file 41598_2021_96093_MOESM2_ESM.tif]

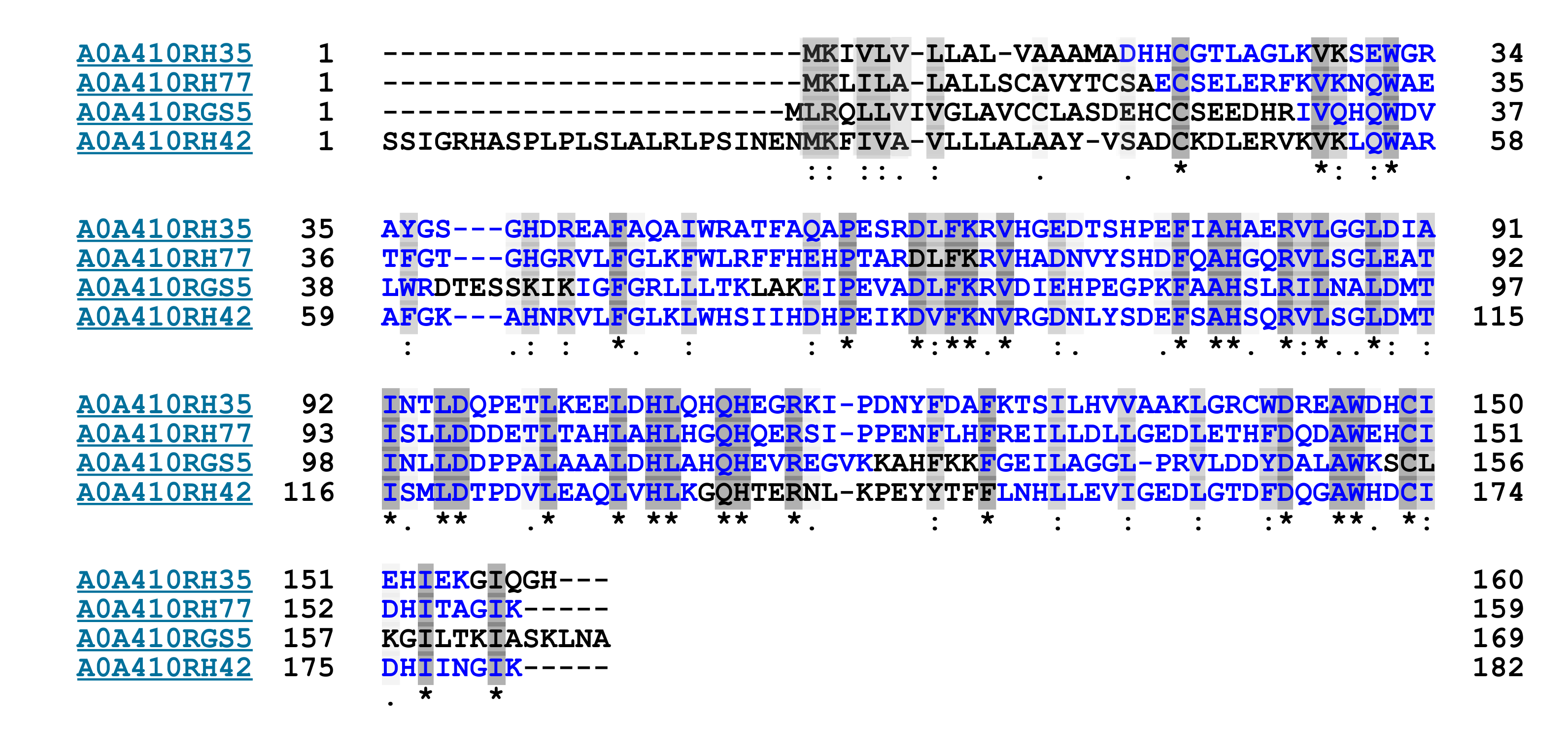

Supplement: Supplementary file 3 — Supplementary Figure 3. [file 41598_2021_96093_MOESM3_ESM.tif]

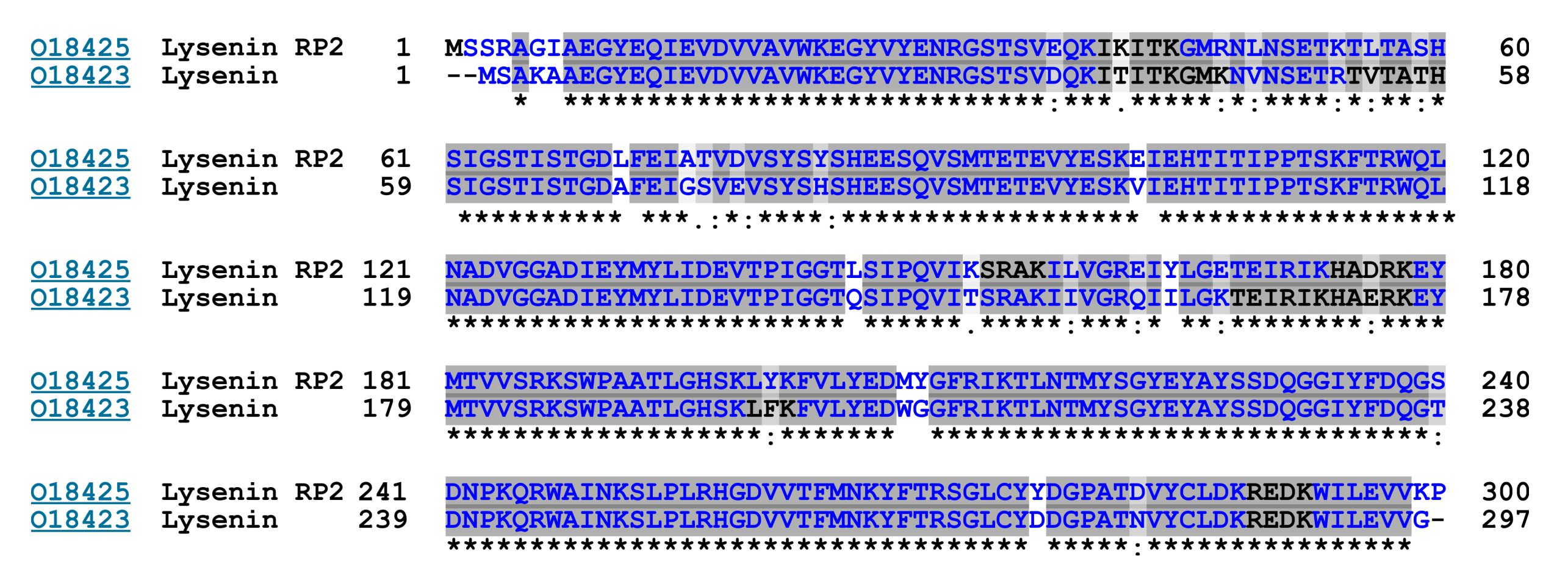

Supplement: Supplementary file 4 — Supplementary Figure 4. [file 41598_2021_96093_MOESM4_ESM.tif]
