# Supplementary material for: Metabolic, structural, and proteomic changes in Candida albicans cells induced by the protein-carbohydrate fraction of Dendrobaena veneta coelomic fluid
Source: Sci Rep. 2021 Aug 18;11:16711. doi: 10.1038/s41598-021-96093-1 (PMC8373886; doi:10.1038/s41598-021-96093-1)
Supplement: Supplementary file 5 — Supplementary Information 5. [file 41598_2021_96093_MOESM5_ESM.docx]

**Supplementary materials**

**Fig. S1.** Analysis of *C. albicans* cells staining with the acridine orange; A- percentage composition of green and red fluorescent cells in samples incubated with different concentration of AAF (25, 50, 100 µg/ml); B- Percentage of cells with red fluorescence in different concentrations of AAF. Significant differences between means are marked with: ***p<0.001.

**Fig.** S2. Analysis of *C. albicans* cell staining with Hoechst and propidium iodide; A- percentage composition of types of cells in samples incubated with different concentration of AAF (25, 50, 100 µg/ml); B- Percentage of unchanged, normal cells incubated with different concentrations of AAF; C- percentage of apoptotic cells incubated with different concentrations of AAF; D- percentage of necrotic cells incubated with different concentrations of AAF. Significant differences between means are marked with: **p<0.01; ***p<0.001.

**Fig. S3**. Comparison of the sequences of two proteins from the group of lysenins identified in the AAF preparation: comparison in Uniprot (sequence alignment). The blue font indicates protein fragments identified by the MS/MS analysis. Lysenin RP2 corresponds to Lysenin-related protein 2.

**Fig. S4.** Comparison of the sequences of four proteins from the group of erythrocruorins identified in the AAF preparation: comparison in Uniprot (sequence alignment). The blue font indicates protein fragments identified by the MS/MS analysis.

**Table. S1**

Results of the basic analysis for *C. albicans* cells with red fluorescence after staining with acridine orange.

**Table S2**

Results of the basic analysis for *C. albicans* cells after staining with Hoechst and propidium iodide.

**Table S3**

Results of Leven’s test and ω^2^ factor for types of *C. albicans* cells after staining with Hoechst and propidium iodide.

**Table S4**

AFF proteins identified in MED-FASP experiments (Peaks Studio software)

**Table S5**

List of proteins identified in the affinity experiment. The AFF fraction was deposited on the NCBr resin and treated with Candida albicans cell lysate (detailed information is provided in the description of Materials and methods of the manuscript). MS/MS analysis was performed for the last millilitre of the column wash (last wash) and proteins dissociated by the elution buffer (elution fraction). Protein identification was carried out in the Peaks Studio software against the *Candida albicans* database.

**Table S6**

Library: List of all proteins identified at 1% FDR in the joint search of DDA measurements in ProteinPilot software used as a spectral library for SWATH-MS experiments.

SWATH-MS: List of all quantified proteins in the SWATH-MS experiments. The values of SWATH-MS intensities after TAS normalization are listed for each measurement. Coefficient of variation was calculated for each protein in each sample using measurement replicates.

C25 to CK, C50 to CK, C100 to CK: Results of t-tests between treated and control samples.
