# Supplementary material for: Metabolic, structural, and proteomic changes in Candida albicans cells induced by the protein-carbohydrate fraction of Dendrobaena veneta coelomic fluid
Source: Sci Rep. 2021 Aug 18;11:16711. doi: 10.1038/s41598-021-96093-1 (PMC8373886; doi:10.1038/s41598-021-96093-1)
Supplement: Supplementary file 6 — Supplementary Tables S1–S3. [file 41598_2021_96093_MOESM6_ESM.docx]

**Table S1.** Results of the basic analysis for *C. albicans* cells with red fluorescence after staining with acridine orange.

| Concentration | Mean | St. Dev. | Shapiro-Wilk test | |
| --- | --- | --- | --- | --- |
|  |  |  | W | p |
| Control culture | 2,34% | 0,004535 | 0,94148 | 0,53336 |
| 25 µg mL^-1^ | 7,71% | 0,003522 | 0,85159 | 0,2447 |
| 50 µg mL^-1^ | 12,10% | 0,006813 | 0,75 | 0 |
| 100 µg mL^-1^ | 21,02% | 0,004371 | 0,92308 | 0,46326 |

**Table S2.** Results of the basic analysis for *C. albicans* cells after staining with Hoechst and propidium iodide.

| Concentration | Cell type | Mean | St. Dev | Shapiro-Wilk test | |
| --- | --- | --- | --- | --- | --- |
|  |  |  |  | W | p |
| Control culture | Normal | 63,96% | 0,069695 | 0,98027 | 0,73014 |
|  | Apoptotic | 10,61% | 0,027497 | 0,91482 | 0,43437 |
|  | Necrotic | 17,44% | 0,022457 | 0,87864 | 0,32042 |
| 25 µg mL^-1^ | Normal | 52,37% | 0,008420 | 0,83633 | 0,20454 |
|  | Apoptotic | 11,56% | 0,007045 | 0,94545 | 0,54976 |
|  | Necrotic | 36,07% | 0,005160 | 0,88353 | 0,33484 |
| 50 µg mL^-1^ | Normal | 30,50% | 0,036806 | 0,98073 | 0,73401 |
|  | Apoptotic | 29,91% | 0,019651 | 0,93981 | 0,52662 |
|  | Necrotic | 39,59% | 0,043962 | 0,90189 | 0,39155 |
| 100 µg mL^-1^ | Normal | 21,38% | 0,039882 | 0,96855 | 0,65951 |
|  | Apoptotic | 22,46% | 0,062598 | 0,84815 | 0,23552 |
|  | Necrotic | 59,50% | 0,031664 | 0,99281 | 0,83787 |

**Table S3.** Results of Leven’s test and ω^2^ factor for types of *C. albicans* cells after staining with Hoechst and propidium iodide.

| Cell type | Leven's test | | ω^2^ |
| --- | --- | --- | --- |
|  | F | p |  |
| Normal | 2,085741 | 0,180521 | 96,22% |
| Apoptotic | 3,010156 | 0,094501 | 82,89% |
| Necrotic | 2,796176 | 0,108868 | 96,22% |
